# Metal-free and solvent-free synthesis of *m*-terphenyls through tandem cyclocondensation of aryl methyl ketones with triethyl orthoformate†

**DOI:** 10.1039/d0ra00578a

**Published:** 2020-03-25

**Authors:** Xiaoqin Xiao, Juan Luo, Zongjie Gan, Wengao Jiang, Qiang Tang

**Affiliations:** College of Pharmacy, Center for Lab Teaching and Management, Chongqing Research Center for Pharmaceutical Engineering, Chongqing Medical University No. 1 Yixueyuan Road Chongqing 400016 P. R. China tangqiang@cqmu.edu.cn

## Abstract

Reported here is a novel cyclocondensation of aryl methyl ketones and triethyl orthoformate for the simple synthesis of *m*-terphenyls. In the presence of a catalytic amount of TfOH, alkyl- and chloro-substituted acetophenones produced a series of terphenyls through a tandem reaction which merged six steps into a one-pot procedure. Moreover, the corresponding ester products were obtained when using other substituted acetophenones as the starting materials under the same reaction conditions.

## Abstract 2

A facile metal-free and solvent-free benzannulation was developed for the construction of *m*-terphenyl derivatives starting from aryl methyl ketones and triethyl orthoformate. This is a tandem reaction which merged six steps into one-pot procedure.

## Graphical abstract

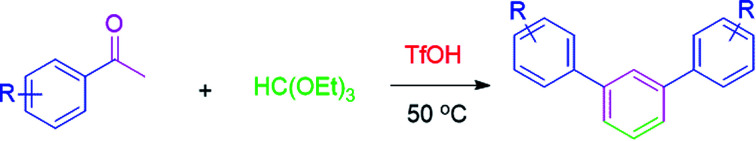

## Introduction

Owing to the amazing photophysical and optical properties, *m*-terphenyls have been extensively applied in materials science, for example in conducting polymers, OLEDs, laser dyes, textile dye carriers, heat storage and transfer agents.^1^ In addition, terphenyl scaffolds have also been found in several naturally occurring compounds with interesting biological properties, like dictyoterphenyl A, trifucol, dunnialol, *etc.*^2^

**Scheme 1:**
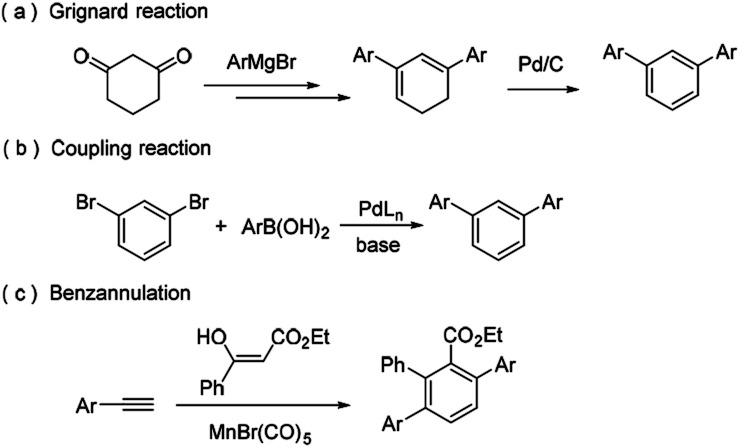
Three types of synthetic strategy to *m*-terphenyls.

The simplest *m*-terphenyl, 1,3-diphenylbenzene, can be synthesized by the addition of the Grignard agent to 1,3-cyclohexanedione (Scheme 1, route a).^3^ To date, various methods have been reported to construct *m*-terphenyl frameworks.^4^ Among these methods, the transition metal-catalyzed coupling reactions have been the most widely used approaches, especially Suzuki–Miyaura cross-coupling reactions (Scheme 1, route b).^5^ Another traditional and superior strategy is benzannulation which can directly assemble acyclic precursors into benzene derivatives.^6^ Various efficient benzannulation methods have been used to construct the terphenyl derivatives^7^ (Scheme 1, route c). However, these methods are generally associated with various drawbacks, which include harsh reaction conditions, expensive metal catalysts, limited substrate scope, and poor chemo- or regioselectivity. Thus, there is still a great demand to develop simple, efficient and regioselective methods for the synthesis of *m*-terphenyls. Herein, we report a novel approach for the facile synthesis of *m*-terphenyls through cyclocondensation reaction of aryl methyl ketones and triethyl orthoformate.^8^

**Scheme 2:**
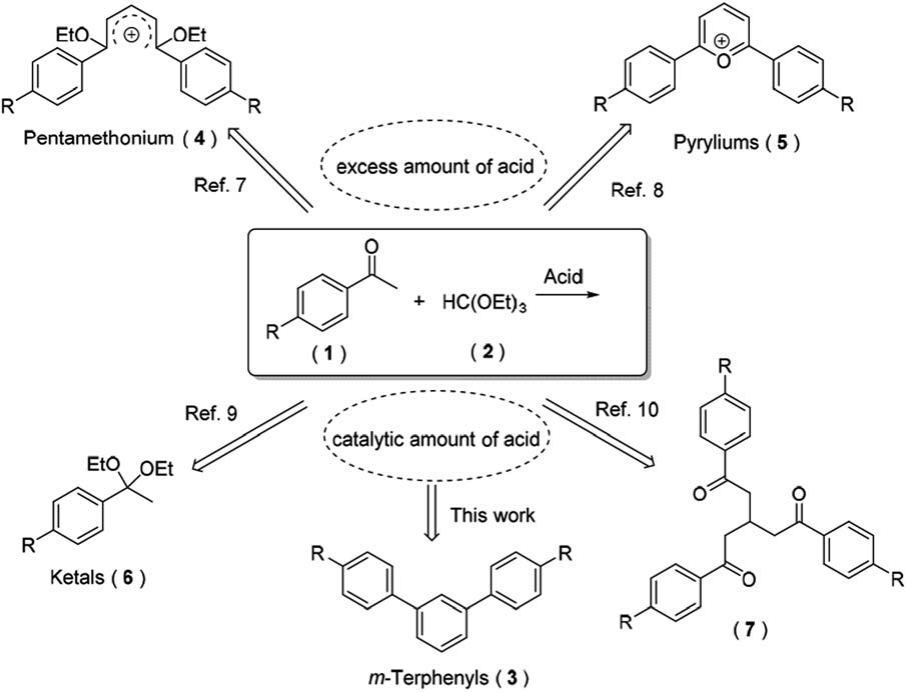
Various products for the reaction of aryl ketones with triethyl orthoformate.

Through an extensive literature research, we found that diverse compounds have been reported as the main product for the reaction of aryl ketones and triethyl orthoformate in the presence of acid under only slightly different conditions (Scheme 2). Open-chain carboxonium ions 4 were obtained when using an equivalent of HClO_4_ or HBF_4_.^9^ However, cyclic carboxonium ions (*i.e.* pyryliums 5) were produced under similar reaction conditions.^10^ Based on their strong fluorescence and high conductivity, carboxonium ions have been frequently employed as functional pigments, phototherapeutic agents and nonlinear optical glasses. Generally, when using trialkyl orthoformates for acetalization of carbonyl compounds in the presence of a catalytic amount of Lewis or Brønsted acid, both ketones and aldehydes could be transformed to their corresponding acetals 6 with excellent yields.^11^ With the aid of microwave and a catalytic amount of boron trifluoride, three equivalents of aryl ketones condensed with one equivalent of triethyl orthoformate to afford the product 7 in moderate yields.^12^ Herein, we surprisingly found that an abnormal tandem cyclocondensation took place under strong acid and an elevated temperature conditions to furnish *m*-terphenyls 3 as its main product.

## Results and discussion

In order to develop an efficient approach to *m*-terphenyls, acetophenone (1a) and triethyl orthoformate (2) were selected as model substrates (Table 1). When we chose three equivalents of trifluoroacetic acid (CF_3_COOH) as catalyst, the reaction proceeded smoothly at 50 °C to furnish a moderate yield of 1,3-diphenylbenzene (3a) (Entry 3). A lower temperature would dramatically reduce the yield of 3a and benefit the formation of carboxonium ions (4 or 5) (Entries 1 and 2), while the self-condensation of 1a would be accelerate to generate the byproduct 1,3,5-triphenylbenzen (8a) at a higher temperature,^13^ thus finally decreased the yield of the desired product (Entry 4). The choice of acids was of considerable importance in this cyclocondensation reaction. In fact, there was a low or no yield of 3a when inorganic acids or weak organic acids were employed (Entries 5–7), and an excess amount of the acid was necessary to guarantee the formation of the desired product (Entries 3 and 8–10). While using strong organic acids, the cyclocondensation reaction with only one equivalent of the acid could produce 3a in moderate yields (Entries 11–12). We believed that large amount of acid would result in the formation of carboxonium ions as byproducts. Therefore catalytic amount of a stronger organic acid was tested. We found that trifluoromethanesulfonic acid (TfOH, 0.2 eq.) led to much cleaner reaction, and finally gave the best yields (Entries 13–17). Surprisingly, we found that the best molar ratio between acetophenone and orthoformate is 1 : 4 which is contradicted with what is dictated by the reaction equation. When reducing the amount of triethyl orthoformate, the yield of 3a was dramatically decreased (Entries 18–19). Moreover, the reactions with different solvents were also examined (Entries 20–23), and yet both the reaction cleanness and the yields were not better than those obtained without any solvent.

**Table 1:** Optimization of reaction conditions^a^

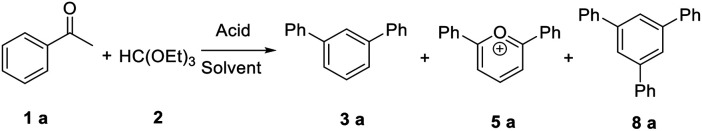					
Entry	Acid	Solvent	*T* (°C)	Time (h)	3a^b^ (%)
1	CF_3_COOH (3.0 eq.)	Neat	0	24	0
2	CF_3_COOH (3.0 eq.)	Neat	25	24	<10
3	CF_3_COOH (3.0 eq.)	Neat	50	2	54
4	CF_3_COOH (3.0 eq.)	Neat	100	1	42
5	CH_3_COOH (3.0 eq.)	Neat	50	24	0
6	—c	Neat	50	24	NA^d^
7	con. HCl (3.0 eq.)	Neat	50	24	0
8	HClO_4_ (3.0 eq.)	Neat	50	2	23
9	HClO_4_ (1.0 eq.)	Neat	50	2	0
10	CF_3_COOH (1.0 eq.)	Neat	50	2	<10
11	TsOH (3.0 eq.)	Neat	50	2	47
12	TsOH (1.0 eq.)	Neat	50	2	56
13	TfOH (3.0 eq.)	Neat	50	2	<10
14	TfOH (2.0 eq.)	Neat	50	2	20
15	TfOH (1.0 eq.)	Neat	50	2	62
16	TfOH (0.2 eq.)	Neat	50	2	81
17	TfOH (0.02 eq.)	Neat	50	2	<10
18	TfOH (0.2 eq)^e^	Neat	50	2	67
19	TfOH (0.2 eq.)^f^	Neat	50	2	<10
20	TfOH (0.2 eq.)	EtOH	50	2	73
21	TfOH (0.2 eq.)	Toluene	50	2	46
22	TfOH (0.2 eq.)	THF	50	2	61
23	TfOH (0.2 eq.)	DCM	50	2	52

a Reagents and conditions: 1a (1.0 mmol), 2 (4.0 mmol).

b Isolated yield.

c Without acid.

d The starting materials were remained.

e 2 (2.0 mmol).

f 2 (1.0 mmol).

With an optimal set of catalysis conditions selected, we were then poised to test the tandem cyclocondensation process and evaluate the substrate scope. When the reaction was conducted at 50 °C in the presence of a catalytic amount of TfOH, we were delighted to discover that different alkyl substituted acetophenones functioned efficiently in the reactions with triethyl orthoformate (Table 2, 1a–1j). Chloro-substituted acetophenones were also effective regardless of the *para*- or *meta*-position on the phenyl ring (Table 2, 1k and 1l). Additionally, simple 2-acetonaphthone was also reacted smoothly to produce 1,3-dinaphthylbenzene in a moderate yield (Table 2, entry 13). It was found that this tandem cyclocondensation reaction was very sensitive to electronic factors as well as steric ones. In fact, most acetophenones with electron donating or withdrawing groups failed to give the desired terphenyl products.

**Table 2:** Synthesis of *m*-teraryls from aryl methyl ketones and triethyl orthoformate^a^

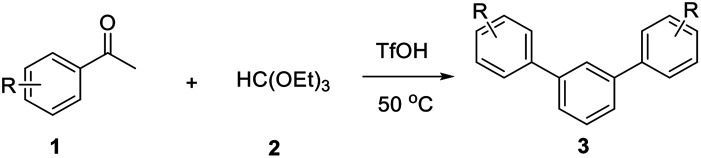			
Entry	Ketone 1	Product 3	Yield^b^ (%)
1	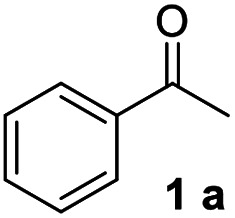	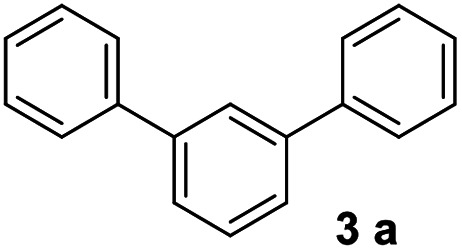	81
2	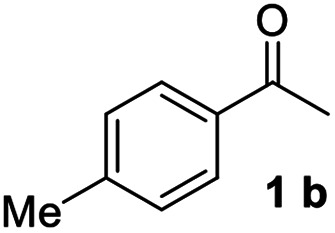	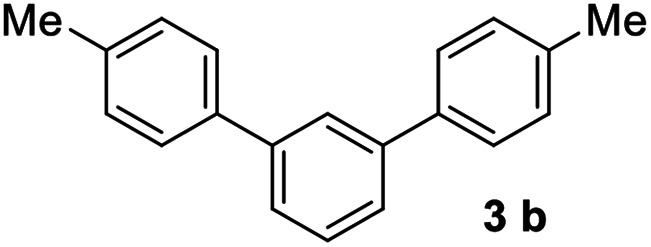	73
3	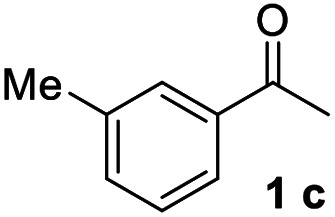	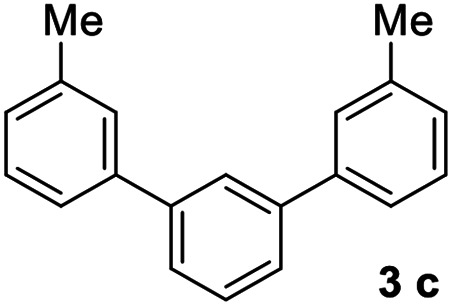	84
4	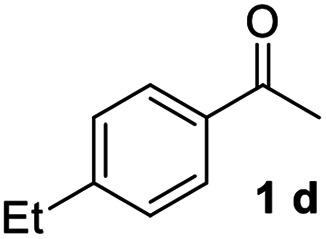	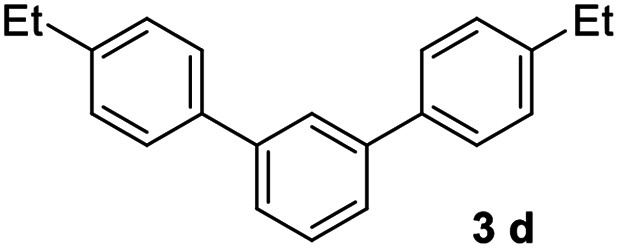	75
5	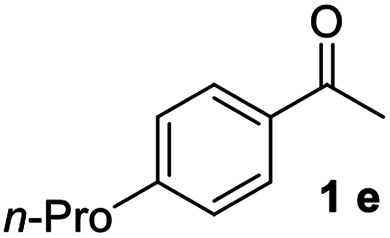	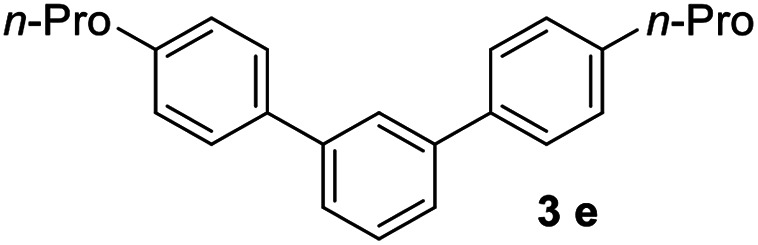	82
6	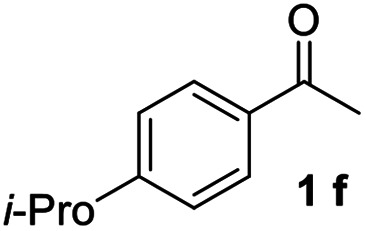	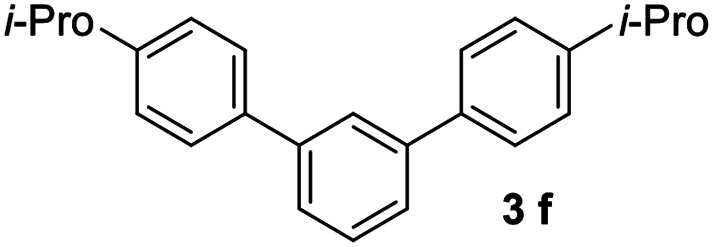	69
7	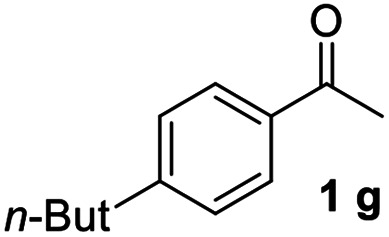	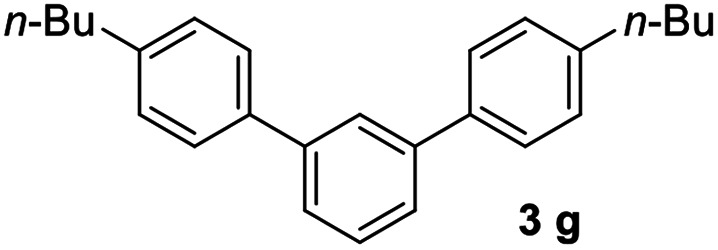	78
8	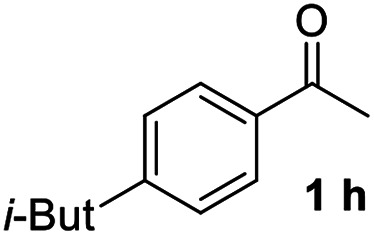	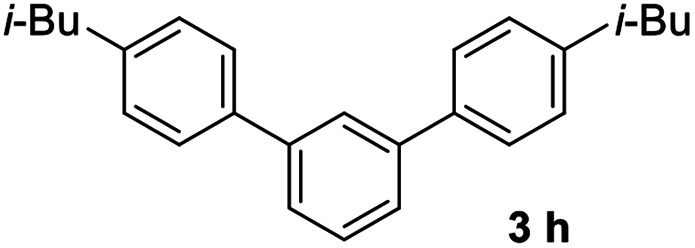	79
9	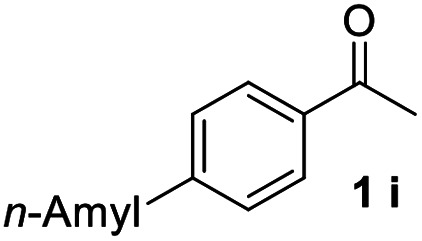	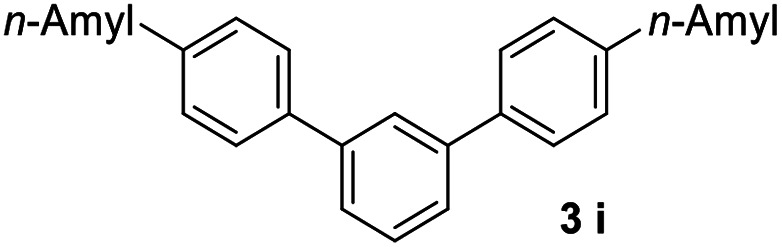	75
10	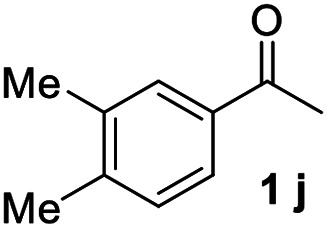	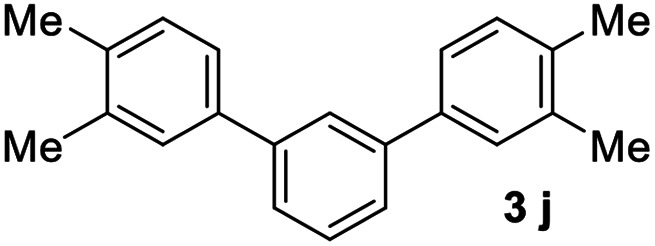	71
11	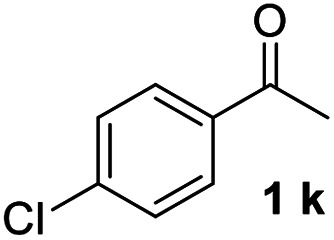	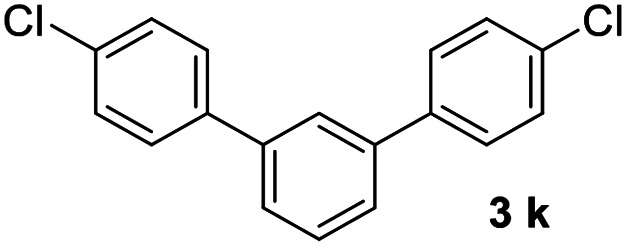	68
12	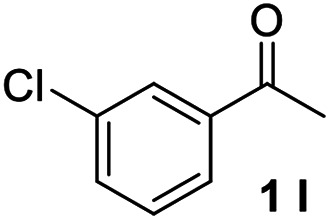	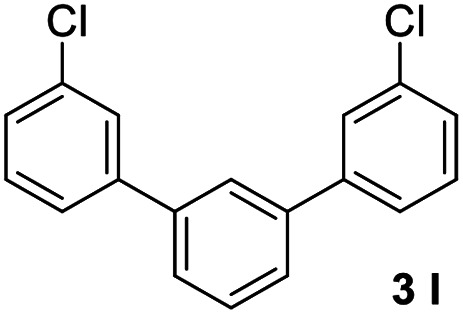	74
13	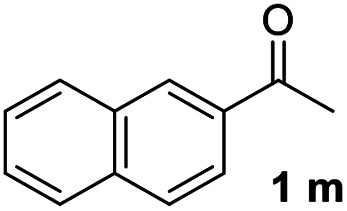	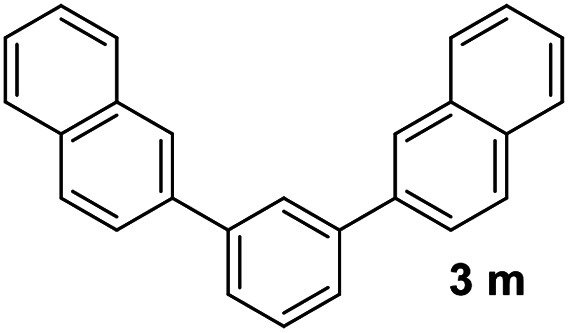	67
14	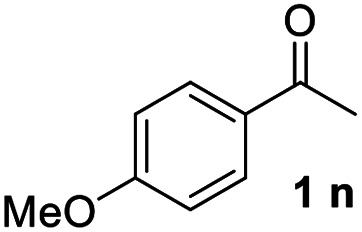	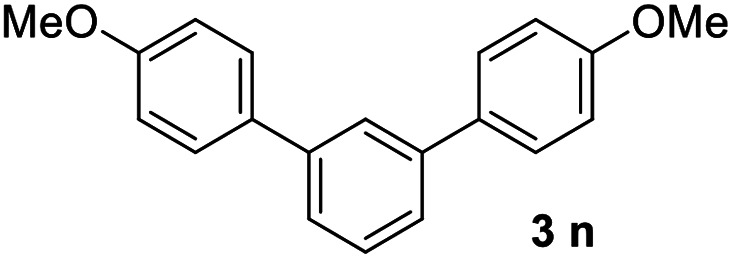	23

a Reagents and conditions: 1 (1.0 mmol), 2 (4.0 mmol), and TfOH (0.1 mmol).

b Isolated yield.

**Scheme 3:**
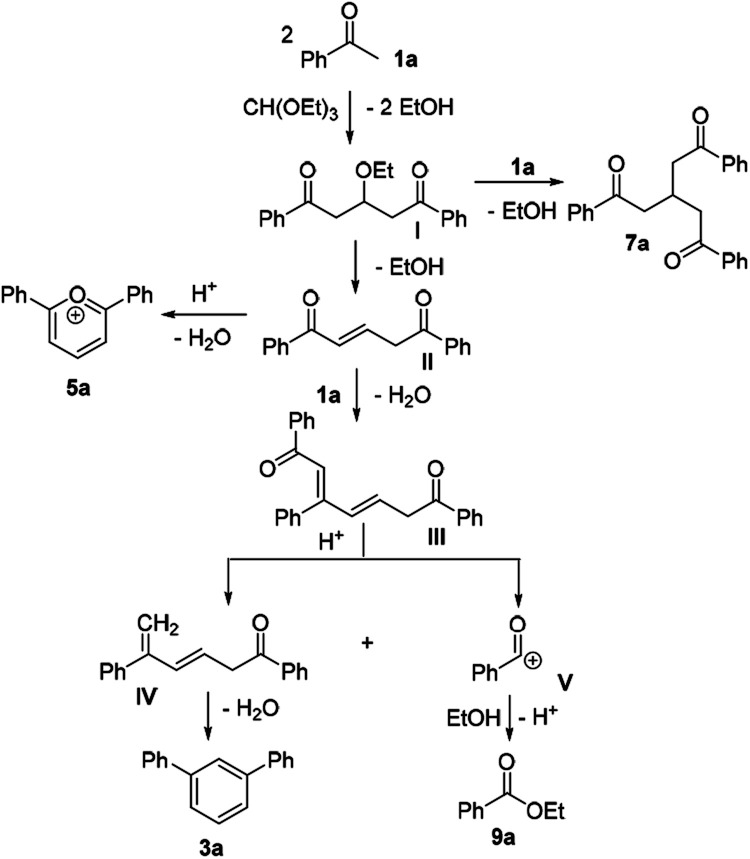
Proposed mechanism.

It was conceived that there should be a complicated reaction pathway for the formation of the desired *m*-terphenyls from aryl methylketones and orthoformate (Scheme 3). And we believed that the departure of a benzoyl ion V from the intermediate III should be the key step.^14^ Therefore it is reasonable that an ester product should be produced in this type of reaction.^15^ To our delight, both terphenyl 3n and ester 9n were obtained for the reaction of *para*-methoxyacetophenone 1n (Table 2, entry 14).

On the basis of our results and the related literatures, a tentative mechanism for the reaction between acetophenone (1a) and triethyl orthoformate under strong acidic conditions is proposed in Scheme 3. Triethyl orthoformate is substituted by two folds of 3a to form an intermediate I, which either undergoes a further substitution reaction to produce compound 7a, or removes of an ethanol molecule to obtain the intermediate II. There are also two pathways for the intermediate II to provide a self-condensed cation 5a and another intermediate III respectively. Under the action of strong acid, the intermediate III is cleaved to provide a benzoyl ion V and an intermediate IV.^14*a*^ The cation V is an active intermediate which can easily react either with ethanol to provide the ester 9a, or with other intermediates to form complex byproducts. While the intermediate IV finally goes through an self-condensation reaction to furnish the terphenyl product 3a. In addition, there may be another proposal to explain the reaction mechanism. The intermediate III proceeds directly through cyclocondensation and benzannulation to afford [1,1′:3′,1′′-terphenyl]-2′-yl(phenyl)methanone or [1,1′:3′,1′′-terphenyl]-4′-yl(phenyl)methanone intermediate, which undergoes decomposition by nucleophiles to generate the final product 3a and the byproduct 9a.

## Conclusions

In summary, a cascade cyclocondensation reaction merged six steps into one-pot procedure has been disclosed. This reaction demonstrates a new protocol for the synthesis of *m*-terphenyls from simple starting materials without any metal catalysts and solvents.

## Experimental section

### General information

Nuclear magnetic resonance spectra (^1^H and ^13^C) were recorded on 600 MHz spectrometers with tetramethylsilane (TMS) as an internal standard. The splitting patterns are designated as singlet (s), doublet (d), triplet (t), quartet (q), dd (doublet of doublets); m (multiplets), and *etc.* All first-order splitting patterns were assigned on the basis of the appearance of the multiplet. Splitting patterns that could not be easily interpreted are designated as multiplet (m) or broad (br). High resolution mass spectral analysis (HRMS) was performed on ESI-QTOP mass spectrometer. Purification was done by column chromatography and preparative TLC using silica gel. TLC analyses were performed on commercial glass plates bearing a 0.25 mm layer of silica gel GF_254_. Visualization was performed using a UV lamp or chemical stains like KMnO_4_ and I_2_. Commercially available materials were used as received.

### General procedure for the reaction between phenol and α-haloketone

To a 25 mL two necked flask under nitrogen atmosphere at 50 °C was added triethyl orthoformate (4.0 mmol) and acetophenones (1.0 mmol). After stirring for 0.5 hour, trifluoromethanesulfonic acid (0.1 mmol) was added into the mixture. After completion of the reaction (monitored by TLC), it was quenched with a saturated sodium carbonate (10 mL), extracted with dichloromethane (3 × 10 mL) and dried with anhydrous sodium sulphate. The organic mixture was concentrated under reduced pressure, and separated by silica-gel column chromatography using ethyl acetate–hexane as eluent in increasing polarity to yield the desired product.

### Characterizations of *m*-teraryls (Table 2)

#### 1,1′:3′,1′′-Terphenyl (3a)^16^

The title compound was obtained as white solid, 81% yield, mp: 83–84 °C, and the analytical data are consistent with those in the literature. ^1^H NMR (600 MHz, CDCl_3_) *δ* 7.83 (t, *J* = 1.7 Hz, 1H), 7.71–7.63 (m, 4H), 7.60 (dd, *J* = 7.1, 1.6 Hz, 2H), 7.53 (dd, *J* = 8.3, 7.0 Hz, 1H), 7.48 (dd, *J* = 10.6, 4.8 Hz, 4H), 7.39 (t, *J* = 7.4 Hz, 2H); ^13^C NMR (151 MHz, CDCl_3_) *δ* 141.82, 141.22, 129.23, 128.84, 127.44, 127.30, 126.20, 126.17.

#### 4,4′′-Dimethyl-1,1′:3′,1′′-terphenyl (3b)^17^

The title compound was obtained as white solid, 73% yield, mp: 125–126 °C, and the analytical data are consistent with those in the literature. ^1^H NMR (600 MHz, CDCl_3_) *δ* 7.78 (s, 1H), 7.59–7.52 (m, 6H), 7.52–7.46 (m, 1H), 7.28 (d, *J* = 7.7 Hz, 4H), 2.42 (s, 6H); ^13^C NMR (151 MHz, CDCl_3_) *δ* 141.64, 138.36, 137.15, 129.50, 129.09, 127.09, 125.77, 125.68, 21.12.

#### 3,3′′-Dimethyl-1,1′:3′,1′′-terphenyl (3c)^17^

The title compound was obtained as colorless oil, 84% yield, and the analytical data are consistent with those in the literature. ^1^H NMR (600 MHz, CDCl_3_) *δ* 7.82 (s, 1H), 7.59 (d, *J* = 7.6 Hz, 2H), 7.55–7.45 (m, 5H), 7.38 (t, *J* = 7.5 Hz, 2H), 7.22 (d, *J* = 7.4 Hz, 2H), 2.47 (s, 6H); ^13^C NMR (151 MHz, CDCl_3_) *δ* 141.82, 141.21, 138.36, 129.05, 128.69, 128.11, 128.05, 126.16, 126.04, 124.36, 21.55.

#### 4,4′′-Diethyl-1,1′:3′,1′′-terphenyl (3d)^17^

The title compound was obtained as colorless crystal, 75% yield, mp: 86–87 °C, and the analytical data are consistent with those in the literature. ^1^H NMR (600 MHz, cdcl_3_) *δ* 7.82 (s, 1H), 7.59 (dd, *J* = 17.9, 7.6 Hz, 6H), 7.54–7.48 (m, 1H), 7.33 (d, *J* = 7.9 Hz, 4H), 2.83–2.63 (m, 4H), 1.32 (t, *J* = 7.6 Hz, 6H); ^13^C NMR (151 MHz, CDCl_3_) *δ* 143.55, 141.71, 138.67, 129.12, 128.36, 127.21, 125.90, 125.75, 28.58, 15.64.

#### 4,4′′-Dipropyl-1,1′:3′,1′′-terphenyl (3e)^18^

The title compound was obtained as white solid, 82% yield, mp: 71–72 °C, and the analytical data are consistent with those in the literature. ^1^H NMR (600 MHz, cdcl_3_) *δ* 7.82 (s, 1H), 7.58 (dd, *J* = 13.5, 7.7 Hz, 6H), 7.55–7.43 (m, 1H), 7.29 (d, *J* = 7.9 Hz, 4H), 2.67 (t, *J* = 7.7 Hz, 4H), 1.72 (dd, *J* = 15.1, 7.5 Hz, 4H), 1.01 (t, *J* = 7.3 Hz, 6H); ^13^C NMR (151 MHz, CDCl_3_) *δ* 141.96, 141.66, 138.61, 129.06, 128.90, 127.06, 125.84, 125.68, 37.71, 24.58, 13.90.

#### 4,4′′-Diisopropyl-1,1′:3′,1′′-terphenyl (3f)

The title compound was obtained as white solid, 69% yield, mp: 99–100 °C. ^1^H NMR (600 MHz, CDCl_3_) *δ* 7.79 (d, *J* = 1.6 Hz, 1H), 7.58 (d, *J* = 8.2 Hz, 4H), 7.56–7.52 (m, 2H), 7.48 (dd, *J* = 8.2, 6.9 Hz, 1H), 7.33 (d, *J* = 8.1 Hz, 4H), 2.97 (dt, *J* = 13.8, 6.9 Hz, 2H), 1.31 (d, *J* = 6.9 Hz, 12H); ^13^C NMR (151 MHz, CDCl_3_) *δ* 148.16, 141.71, 138.84, 129.12, 127.23, 126.94, 125.95, 125.79, 33.88, 24.09; HRMS (ESI) calcd for C_18_H_13_ (M + H)^+^: 229.1012, found: 229.1010.

#### 4,4′′-Dibutyl-1,1′:3′,1′′-terphenyl (3g)^19^

The title compound was obtained as yellowish solid, 78% yield, mp: 47–48 °C, and the analytical data are consistent with those in the literature. ^1^H NMR (600 MHz, CDCl_3_) *δ* 7.81 (d, *J* = 1.4 Hz, 1H), 7.62–7.53 (m, 6H), 7.52–7.44 (m, 1H), 7.29 (d, *J* = 7.8 Hz, 4H), 2.68 (t, *J* = 7.8 Hz, 4H), 1.74–1.61 (m, 4H), 1.42 (dd, *J* = 14.9, 7.4 Hz, 4H), 0.97 (t, *J* = 7.4 Hz, 6H); ^13^C NMR (151 MHz, CDCl_3_) *δ* 142.21, 141.70, 138.60, 129.10, 128.89, 127.11, 125.87, 125.71, 35.35, 33.70, 22.46, 14.02.

#### 4,4′′-Diisobutyl-1,1′:3′,1′′-terphenyl (3h)

The title compound was obtained as white solid, 79% yield, mp: 67–68 °C. ^1^H NMR (600 MHz, CDCl_3_) *δ* 7.83 (d, *J* = 1.5 Hz, 1H), 7.58 (dd, *J* = 13.9, 4.9 Hz, 6H), 7.55–7.44 (m, 1H), 7.33–7.18 (m, 4H), 2.55 (d, *J* = 7.2 Hz, 4H), 1.94 (dt, *J* = 13.5, 6.8 Hz, 2H), 0.97 (d, *J* = 6.6 Hz, 12H); ^13^C NMR (151 MHz, CDCl_3_) *δ* 141.68, 141.00, 138.63, 129.60, 129.11, 126.95, 125.86, 125.70, 45.14, 30.31, 22.47; HRMS (ESI) calcd for C_26_H_31_ (M + H)^+^: 343.2420, found: 343.2425.

#### 4,4′′-Dipentyl-1,1′:3′,1′′-terphenyl (3i)^17^

The title compound was obtained as white solid, 75% yield, mp: 56–57 °C, and the analytical data are consistent with those in the literature. ^1^H NMR (600 MHz, CDCl_3_) *δ* 7.81 (s, 1H), 7.61–7.53 (m, 6H), 7.49 (dd, *J* = 8.2, 7.1 Hz, 1H), 7.29 (d, *J* = 8.0 Hz, 4H), 2.78–2.55 (m, 4H), 1.75–1.64 (m, 4H), 1.47–1.32 (m, 8H), 0.93 (t, *J* = 6.8 Hz, 6H); ^13^C NMR (151 MHz, CDCl_3_) *δ* 142.25, 141.69, 138.60, 129.10, 128.88, 127.11, 125.87, 125.71, 35.64, 31.61, 31.24, 22.61, 14.09.

#### 3,3′′,4,4′′-Tetramethyl-1,1′:3′,1′′-terphenyl (3j)

The title compound was obtained as yellowish oil, 71% yield, mp: 57–58 °C. ^1^H NMR (600 MHz, CDCl_3_) *δ* 7.78 (t, *J* = 1.7 Hz, 1H), 7.56–7.51 (m, 2H), 7.50–7.46 (m, 1H), 7.44 (s, 2H), 7.40 (dd, *J* = 7.7, 1.8 Hz, 2H), 7.24 (d, *J* = 7.7 Hz, 2H), 2.36 (s, 6H), 2.33 (s, 6H); ^13^C NMR (151 MHz, CDCl_3_) *δ* 141.73, 138.91, 136.93, 135.79, 130.07, 128.99, 128.53, 125.82, 125.63, 124.60, 19.94, 19.45; HRMS (ESI) calcd for C_22_H_23_ (M + H)^+^: 287.1794, found: 287.1791.

#### 4,4′′-Dichloro-1,1′:3′,1′′-terphenyl (3k)^20^

The title compound was obtained as white solid, 68% yield, mp: 112–113 °C, and the analytical data are consistent with those in the literature. ^1^H NMR (600 MHz, CDCl_3_) *δ* 7.71 (t, *J* = 1.5 Hz, 1H), 7.59–7.49 (m, 7H), 7.47–7.40 (m, 4H); HRMS (ESI) calcd for C_18_H_13_Cl_2_ (M + H)^+^: 299.0389, found: 299.0388.

#### 3,3′′-Dichloro-1,1′:3′,1′′-terphenyl (3l)^21^

The title compound was obtained as yellowish oil, 74% yield, and the analytical data are consistent with those in the literature. ^1^H NMR (600 MHz, CDCl_3_) *δ* 7.73 (s, 1H), 7.63 (s, 2H), 7.57 (d, *J* = 6.8 Hz, 2H), 7.53 (dd, *J* = 10.2, 7.7 Hz, 3H), 7.40 (t, *J* = 7.8 Hz, 2H), 7.38–7.33 (m, 2H); ^13^C NMR (151 MHz, CDCl_3_) *δ* 142.77, 140.58, 134.78, 130.10, 129.50, 127.57, 127.39, 126.65, 126.01, 125.42.

#### 1,3-Di(naphthalen-2-yl)benzene (3m)^22^

The title compound was obtained as white solid, 67% yield, mp: 144–145 °C, and the analytical data are consistent with those in the literature. ^1^H NMR (600 MHz, CDCl_3_) *δ* 8.15 (s, 2H), 8.08 (s, 1H), 7.96 (dd, *J* = 14.4, 8.2 Hz, 4H), 7.91 (d, *J* = 7.7 Hz, 2H), 7.86 (d, *J* = 8.4 Hz, 2H), 7.76 (d, *J* = 7.6 Hz, 2H), 7.62 (t, *J* = 7.6 Hz, 1H), 7.58–7.48 (m, 4H); ^13^C NMR (151 MHz, CDCl_3_) *δ* 141.80, 138.50, 133.69, 132.71, 129.38, 128.51, 128.23, 127.67, 126.64, 126.48, 126.36, 126.02, 125.97, 125.65.

#### 4,4′′-Dimethoxy-1,1′:3′,1′′-terphenyl (3n)^17^

The title compound was obtained as white solid, 23% yield, mp: 193–194 °C, and the analytical data are consistent with those in the literature. ^1^H NMR (600 MHz, CDCl_3_) *δ* 7.72 (d, *J* = 1.6 Hz, 1H), 7.63–7.54 (m, 4H), 7.54–7.40 (m, 3H), 7.05–6.96 (m, 4H), 3.87 (s, 6H); ^13^C NMR (151 MHz, CDCl_3_) *δ* 159.21, 141.32, 133.81, 129.11, 128.26, 125.33, 125.15, 114.22, 55.36.

### Characterizations of other byproducts

#### 3-(2-Oxo-2-phenylethyl)-1,5-diphenylpentane-1,5-dione (7a)^12*a*^

The title compound was obtained as white solid, mp: 139–140 °C, and the analytical data are consistent with those in the literature. ^1^H NMR (600 MHz, CDCl_3_) *δ* 8.01 (d, *J* = 7.8 Hz, 6H), 7.55 (t, *J* = 7.3 Hz, 3H), 7.45 (t, *J* = 7.7 Hz, 6H), 3.44–3.31 (m, 1H), 3.26 (d, *J* = 6.4 Hz, 6H); ^13^C NMR (151 MHz, CDCl_3_) *δ* 199.49, 136.81, 133.21, 128.63, 128.21, 42.39, 27.66.

#### 5′-Phenyl-1,1′:3′,1′′-terphenyl (8a)^13*b*^

The title compound was obtained as white solid, mp: 170–171 °C, and the analytical data are consistent with those in the literature. ^1^H NMR (600 MHz, CDCl_3_) *δ* 7.80 (s, 3H), 7.72 (d, *J* = 8.0 Hz, 6H), 7.49 (t, *J* = 7.6 Hz, 6H), 7.40 (t, *J* = 7.3 Hz, 3H); ^13^C NMR (151 MHz, CDCl_3_) *δ* 142.35, 141.16, 128.85, 127.55, 127.36, 125.18.

### Characterizations of esters

#### Ethyl 4-methoxybenzoate (9n)^23^

The title compound was obtained as colorless oil, 46% yield, and the analytical data are consistent with those in the literature. ^1^H NMR (600 MHz, CDCl_3_) *δ* 8.00 (d, *J* = 8.9 Hz, 2H), 6.91 (d, *J* = 8.9 Hz, 2H), 4.35 (q, *J* = 7.1 Hz, 2H), 3.86 (s, 3H), 1.38 (t, *J* = 7.1 Hz, 3H); ^13^C NMR (151 MHz, CDCl_3_) *δ* 166.43, 163.25, 131.55, 122.97, 113.55, 60.66, 55.43, 14.40.

## Conflicts of interest

There are no conflicts to declare.

## Supplementary Material

RA-010-D0RA00578A-s001

## References

[cit1] Lee C. W., Lee J. Y. (2014). Org. Electron..

[cit2] Kikuchi H., Matsuo Y., Katou Y., Kubohara Y., Oshima Y. (2012). Tetrahedron.

[cit3] Woods G. F., Tucker I. W. (1948). J. Am. Chem. Soc..

[cit4] Shetgaonkar S. E., Singh F. V. (2019). Synth. Commun..

[cit5] Antelo Miguez J. M., Adrio L. A., Sousa-Pedrares A., Vila J. M., Hii K. K. (2007). J. Org. Chem..

[cit6] Poudel T. N., Tamargo R. J. I., Cai H., Lee Y. R. (2018). Asian J. Org. Chem..

[cit7] Saito S., Yamamoto Y. (2000). Chem. Rev..

[cit8] Kovalev A. I., Lyakhovetskli Y. I., Teplyakov M. M., Rusanov A. L., Petrovskii P. V., Yakushin S. O. (1993). Russ. Chem. Bull..

[cit9] Obaya N., Payrastre C., Madaule Y. (2001). Tetrahedron.

[cit10] Hong Y.-M., Kim H.-O., Kim K.-H., Cho S.-I. (2012). Journal of Sensor Science and Technology.

[cit11] Fife T. H., Jao L. K. (1965). J. Org. Chem..

[cit12] Yu J., Hu Y., Huang Q., Ma R., Yang S. (2000). Synth. Commun..

[cit13] Sasaki Y., Pittman C. U. (1973). J. Org. Chem..

[cit14] Elderfield R. C., King T. P. (1954). J. Am. Chem. Soc..

[cit15] Justik M. W. (2007). Tetrahedron Lett..

[cit16] Huang K., Ke X., Wang H., Wang J., Zhou C., Xu X., Liu L., Li J. (2015). Org. Biomol. Chem..

[cit17] Teng Q., Mo S., Pan J., Wu N., Wang H., Pan Y. (2016). Synthesis.

[cit18] Gu N., Liu Y., Liu P., Ma X., Yan L., Dai B. (2015). Chin. J. Chem..

[cit19] Igarashi T., Haito A., Chatani N., Tobisu M. (2018). ACS Catal..

[cit20] Córsico E. F., Rossi R. A. (2002). J. Org. Chem..

[cit21] Ranjani G., Nagarajan R. (2017). Org. Lett..

[cit22] Mohamed R. K., Mondal S., Gold B., Evoniuk C. J., Banerjee T., Hanson K., Alabugin I. V. (2015). J. Am. Chem. Soc..

[cit23] Salvadori J., Balducci E., Zaza S., Petricci E., Taddei M. (2010). J. Org. Chem..

