# Supplementary material for: Metal-free and solvent-free synthesis of m-terphenyls through tandem cyclocondensation of aryl methyl ketones with triethyl orthoformate
Source: RSC Adv. 2020 Mar 25;10(20):12113–8. doi: 10.1039/d0ra00578a (PMC9050746; doi:10.1039/d0ra00578a)
Supplement: RA-010-D0RA00578A-s001 [file RA-010-D0RA00578A-s001.pdf]

Electronic Supplementary Material (ESI) for RSC Advances.
This journal is © The Royal Society of Chemistry 2020

Supporting Information

Metal-free and Solvent-free Synthesis of *m*-Terphenyls through Tandem Cyclocondensation of Aryl Methyl Ketones with Triethyl Orthoformate

Xiaoqin Xiao,[a] Juan Luo,[a] Zongjie Gan,[a] Wengao Jiang [a] and Qiang Tang

College of Pharmacy, Center for Lab Teaching and Management, Chongqing Research Center for Pharmaceutical Engineering; Chongqing Medical University. No.1 Yixueyuan Road, Chongqing 400016, P. R. China.

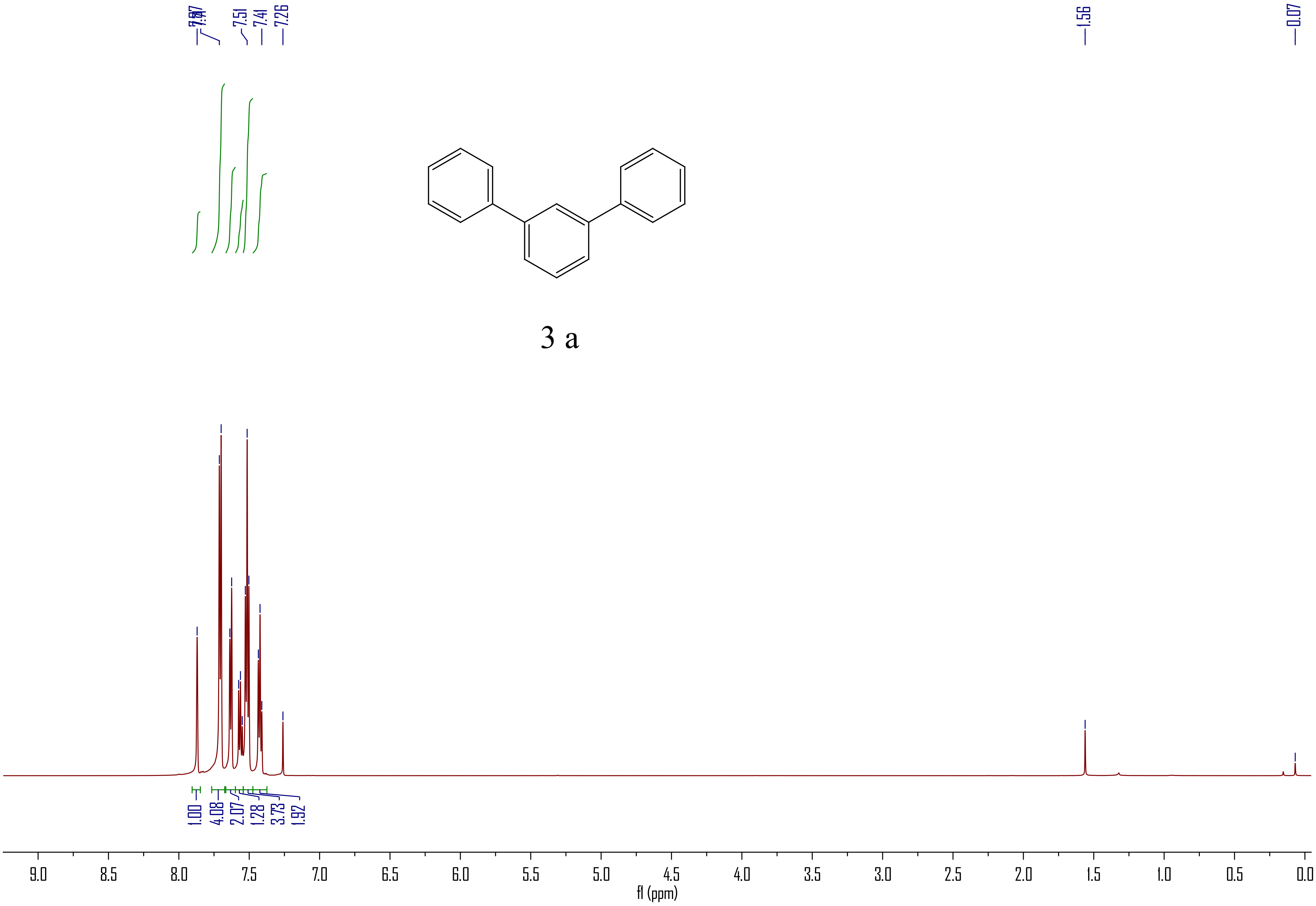
3 a
7.87
7.51
7.41
7.26
1.56
0.07
1.00
4.08
2.07
1.28
3.73
1.92
9.0 8.5 8.0 7.5 7.0 6.5 6.0 5.5 5.0 4.5 4.0 3.5 3.0 2.5 2.0 1.5 1.0 0.5 0.0
fl (ppm)

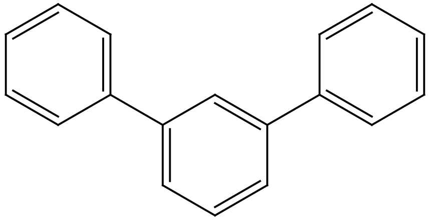

3 a

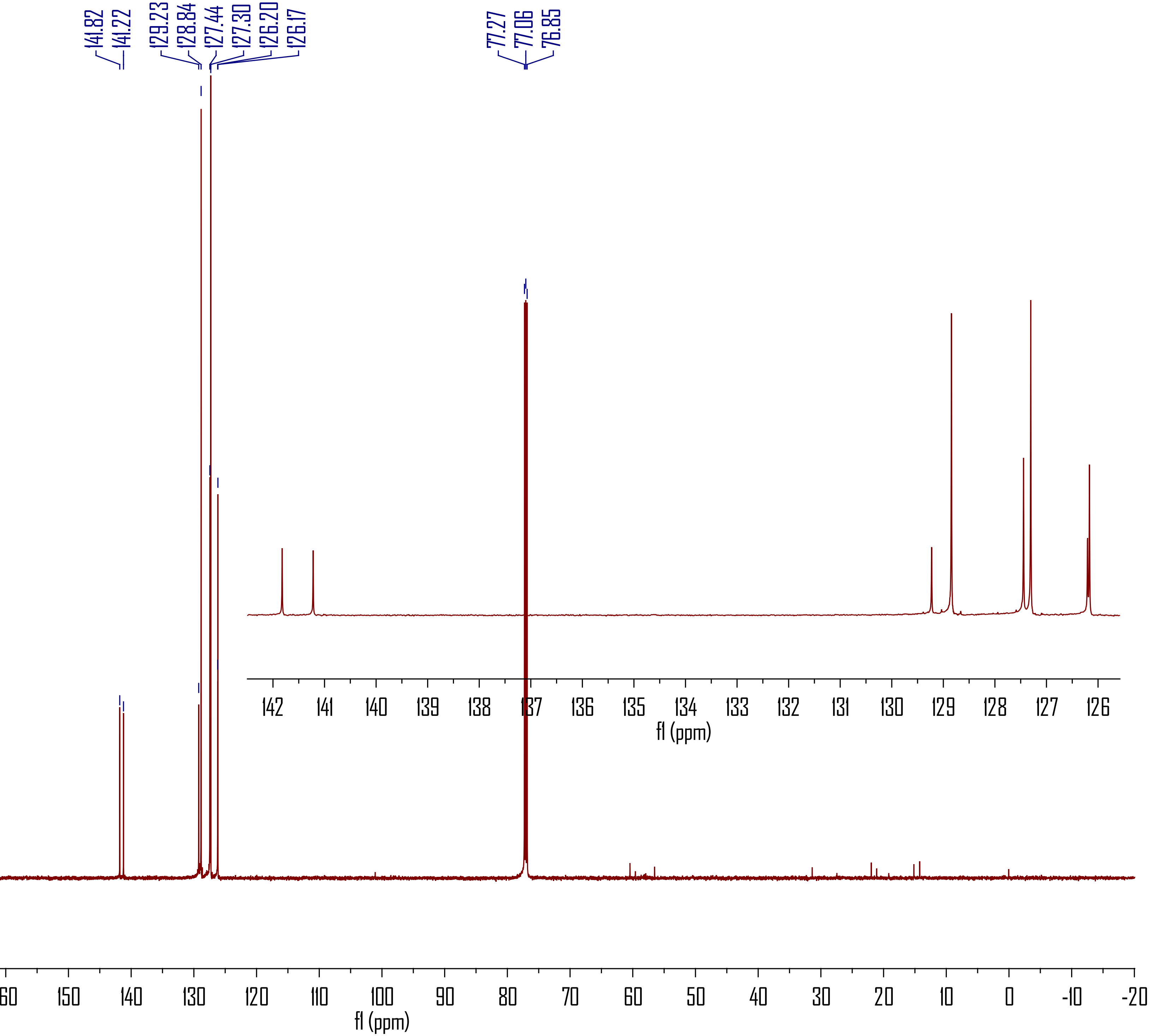
141.82
141.22
129.23
128.84
127.44
127.30
126.20
126.17
77.27
77.06
76.85
142 141 140 139 138 137 136 135 134 133 132 131 130 129 128 127 126
f1 (ppm)
220 210 200 190 180 170 160 150 140 130 120 110 100 90 80 70 60 50 40 30 20 10 0 -10 -20
f1 (ppm)

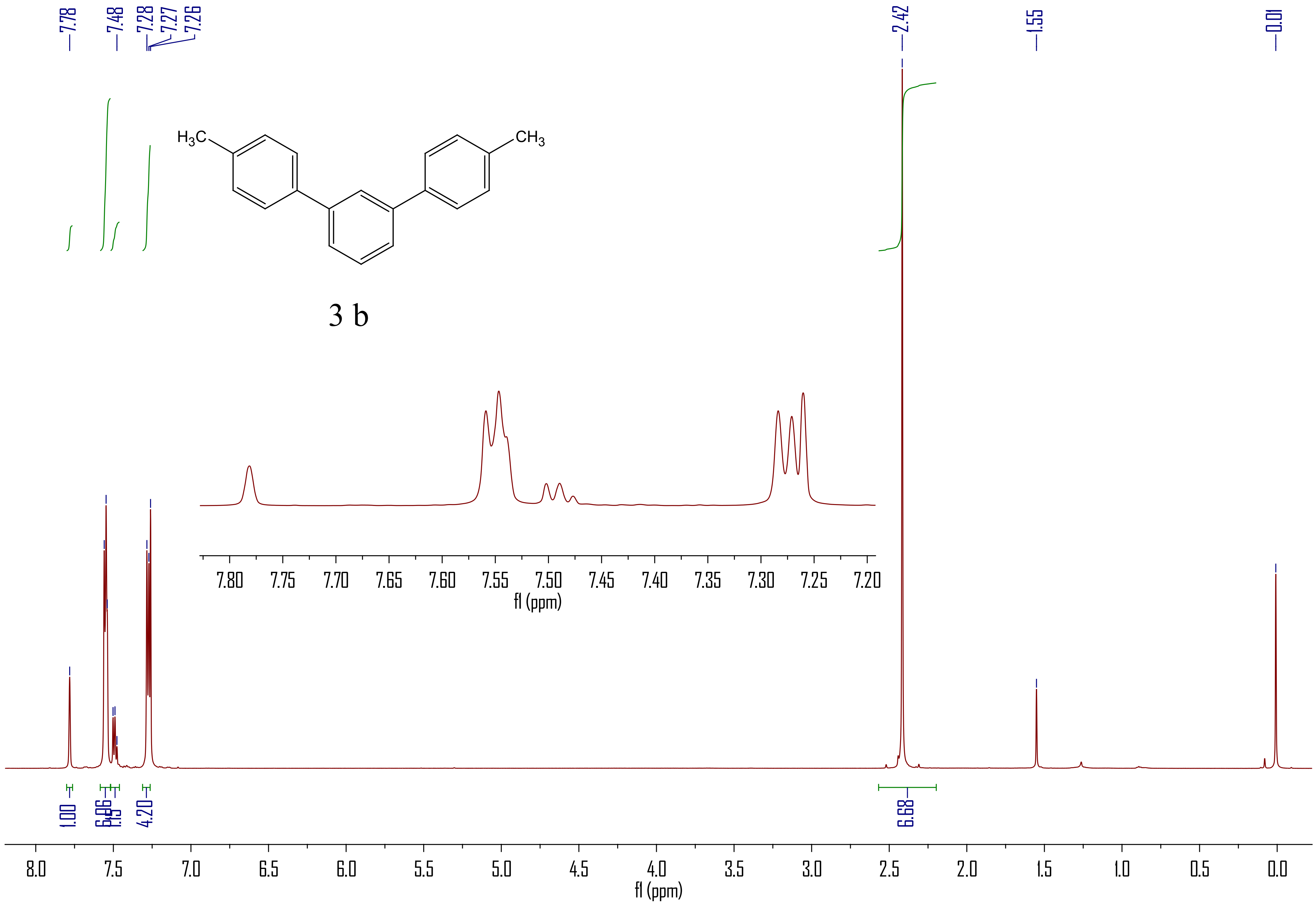
7.78
7.48
7.28
7.27
7.26
2.42
1.55
0.01
H_3C
CH_3
3 b
1.00
4.20
6.68
fl (ppm)
fl (ppm)

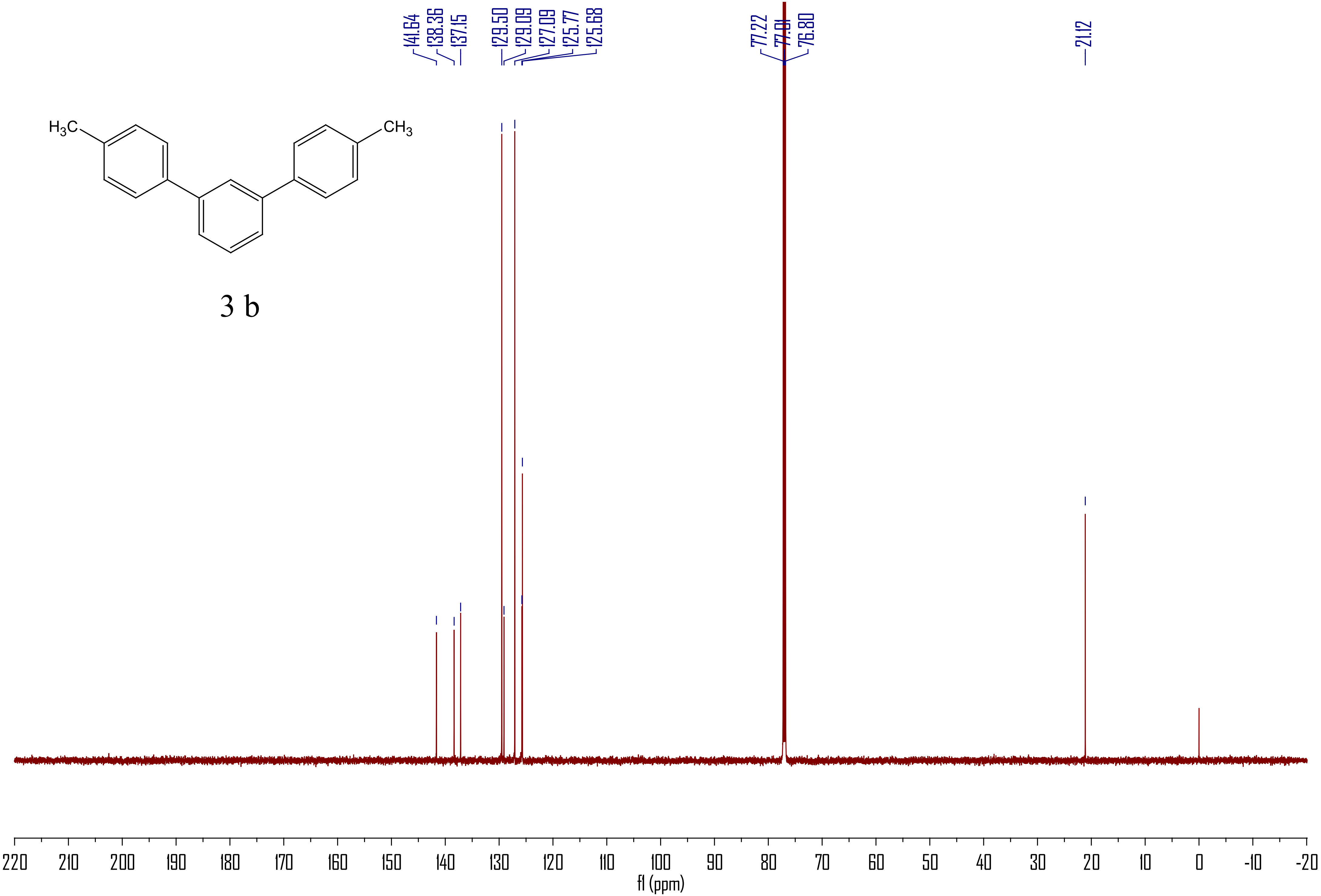
141.64
138.36
137.15
129.50
129.09
127.09
125.77
125.68
77.22
77.01
76.80
21.12
H_3C
CH_3
3 b
220 210 200 190 180 170 160 150 140 130 120 110 100 90 80 70 60 50 40 30 20 10 0 -10 -20
f1 (ppm)

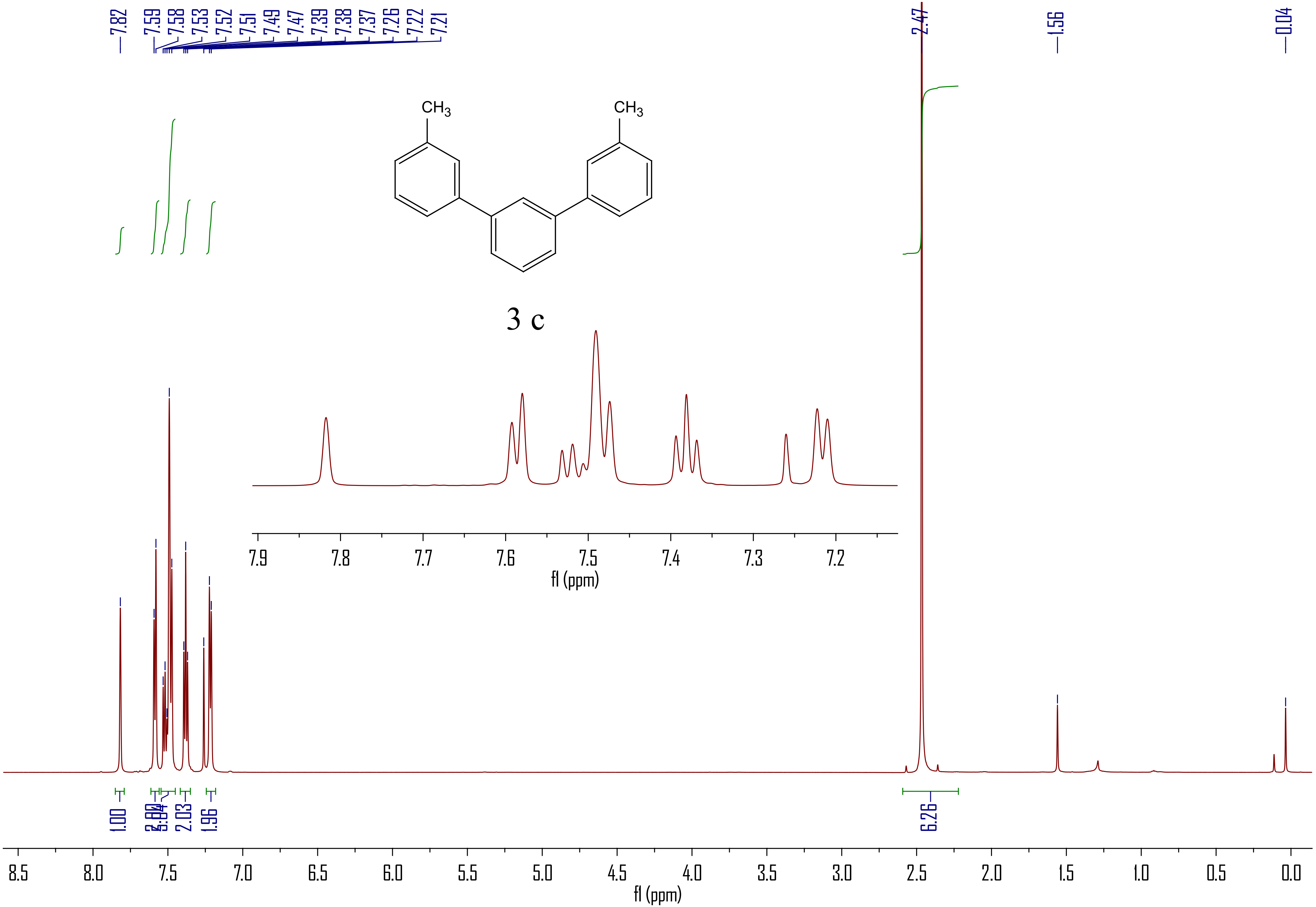
7.82
7.59
7.58
7.53
7.52
7.51
7.49
7.47
7.39
7.38
7.37
7.26
7.22
7.21
2.47
1.56
0.04
CH3
CH3
3 c
fl (ppm)
1.00
2.03
1.96
6.26
fl (ppm)

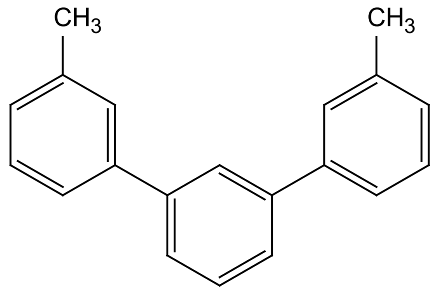

CH3
CH3

3 c

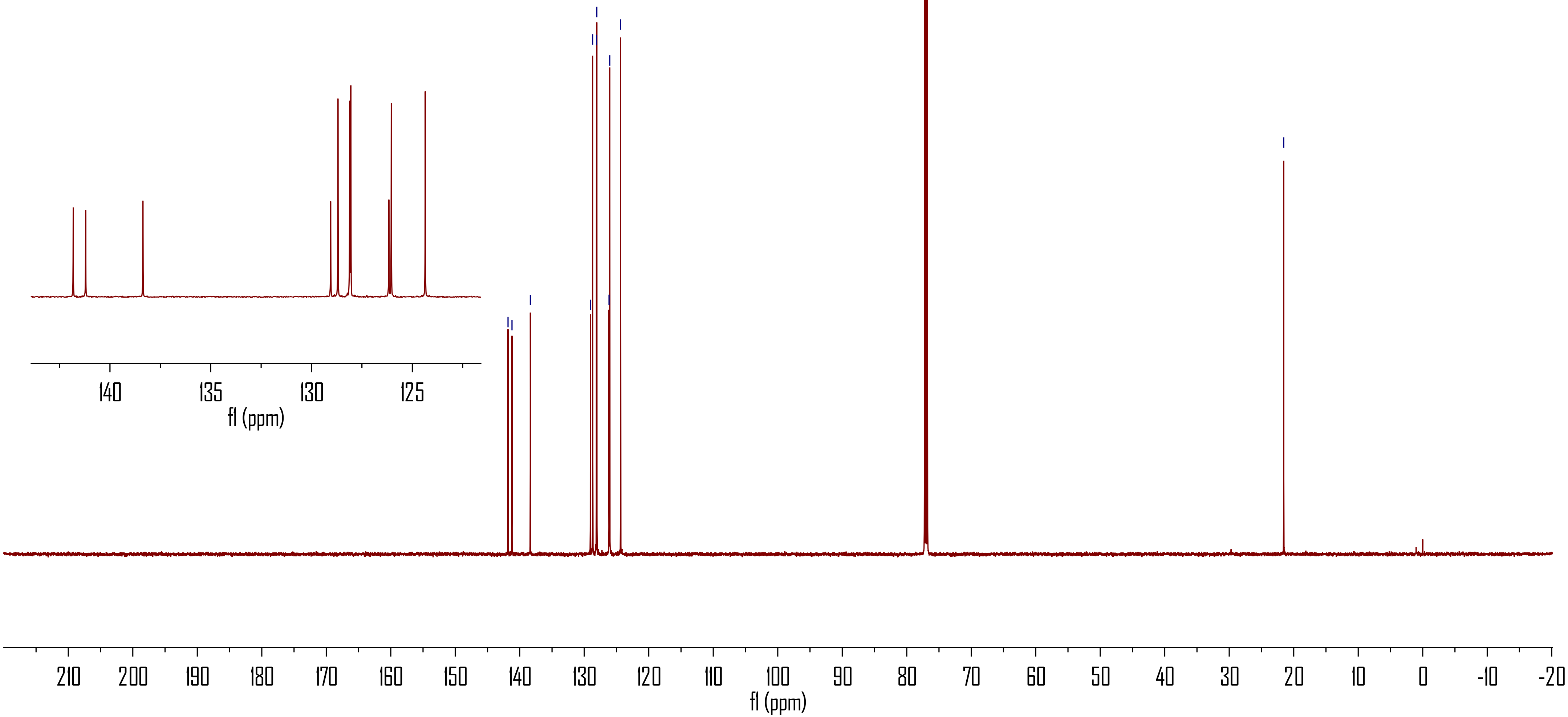

141.82
141.21
138.36
129.05
128.69
128.11
128.05
126.16
126.04
124.36
77.22
77.01
76.80
21.55
140
135
130
125
f1 (ppm)
210
200
190
180
170
160
150
140
130
120
110
100
90
80
70
60
50
40
30
20
10
0
-10
-20
f1 (ppm)

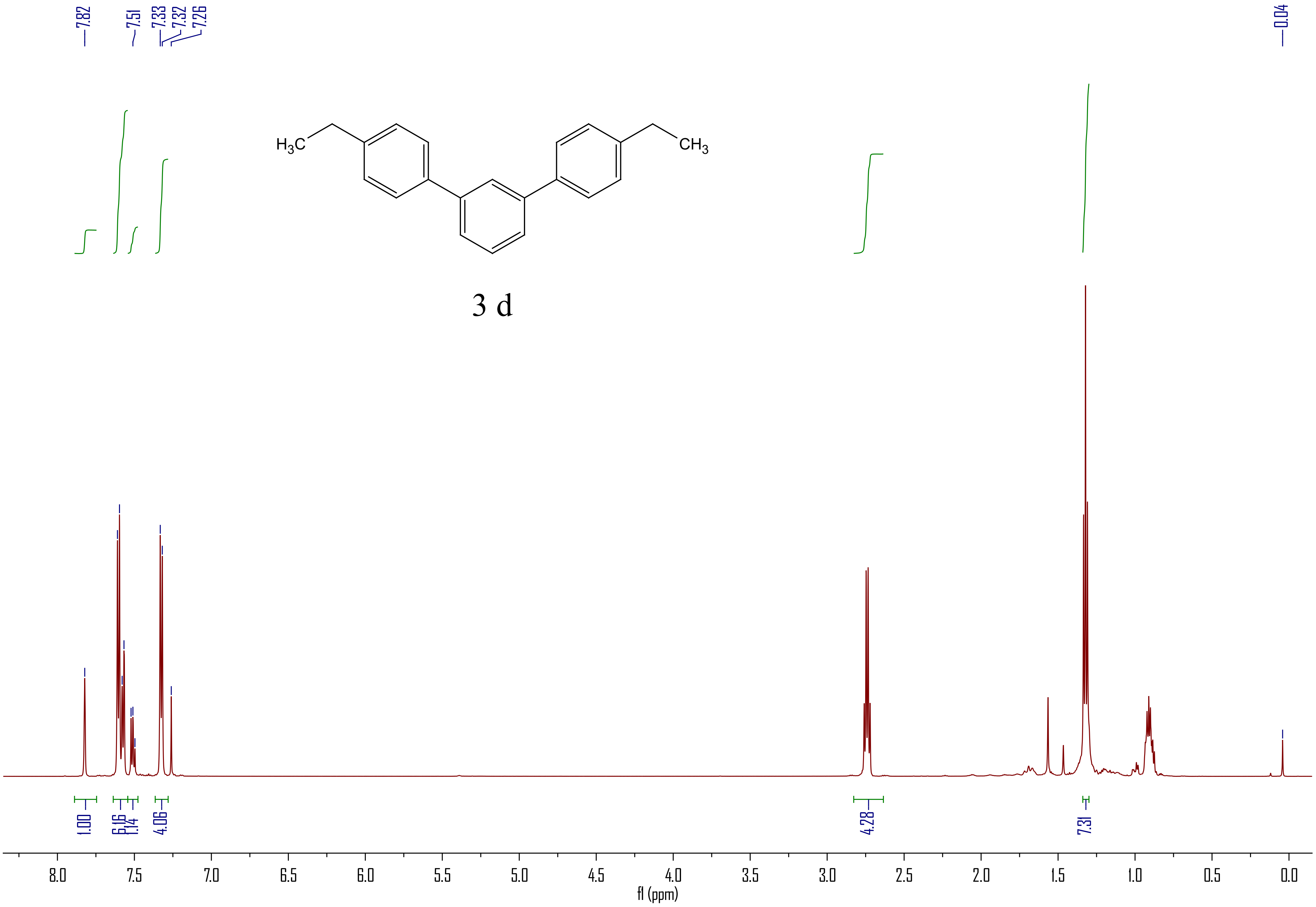
7.82
7.51
7.33
7.32
7.26
0.04
H_3C
CH_3
3 d
1.00
6.16
1.14
4.06
4.28
7.31
8.0 7.5 7.0 6.5 6.0 5.5 5.0 4.5 4.0 3.5 3.0 2.5 2.0 1.5 1.0 0.5 0.0
f1 (ppm)

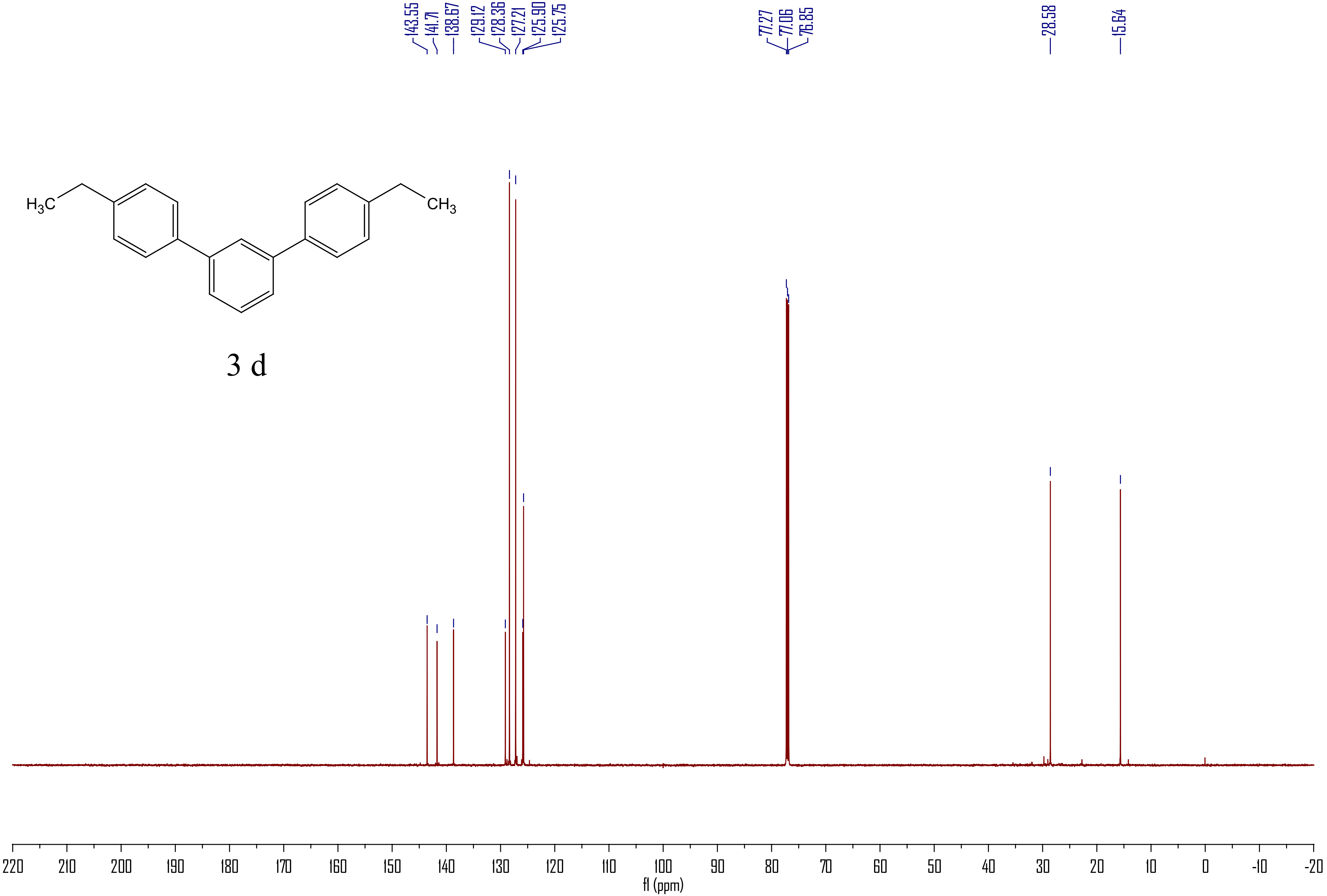
143.55
141.71
138.67
129.12
128.36
127.21
125.90
125.75
77.27
77.06
76.85
28.58
15.64
H_3C
CH_3
220 210 200 190 180 170 160 150 140 130 120 110 100 90 80 70 60 50 40 30 20 10 0 -10 -20
f1 (ppm)

3 d

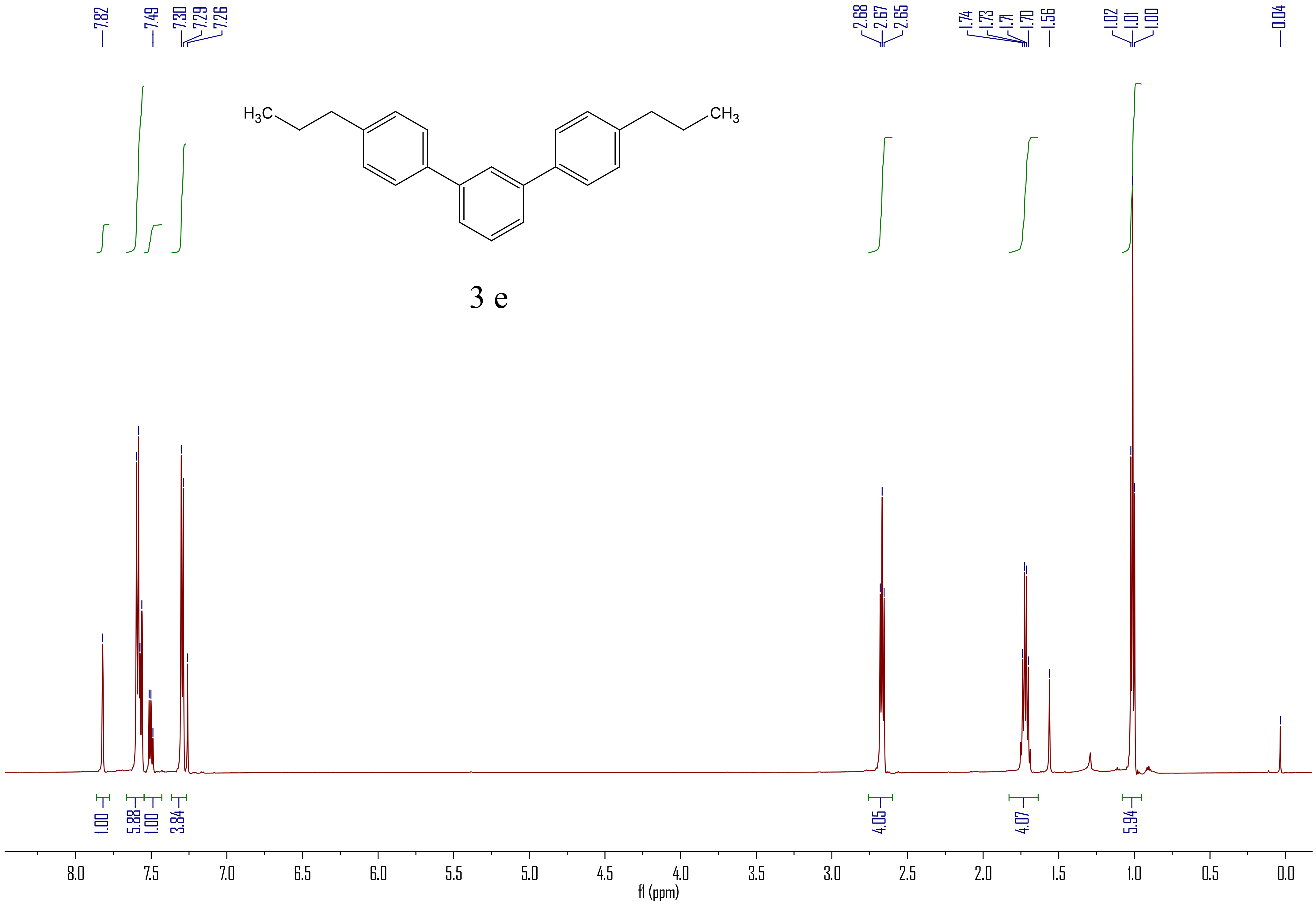
7.82
7.49
7.30
7.29
7.26
2.68
2.67
2.65
1.74
1.73
1.71
1.70
1.56
1.02
1.01
1.00
0.04
H3C
CH3
3 e
1.00
5.88
1.00
3.84
4.05
4.07
5.94
8.0
7.5
7.0
6.5
6.0
5.5
5.0
4.5
4.0
3.5
3.0
2.5
2.0
1.5
1.0
0.5
0.0
f1 (ppm)

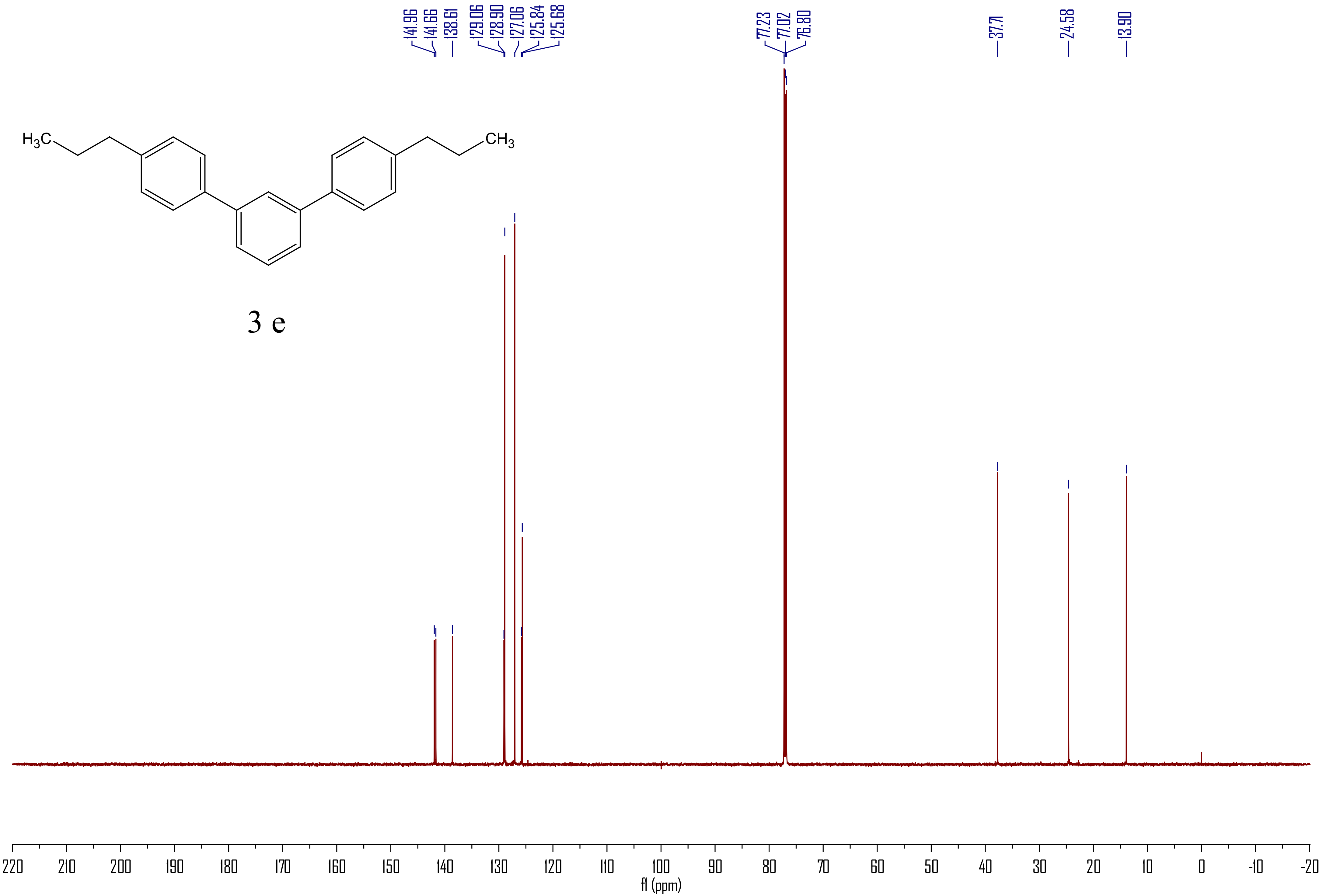
H3C
CH3
3 e
141.96
141.66
138.61
129.06
128.90
127.06
125.84
125.68
77.23
77.02
76.80
37.71
24.58
13.90
220 210 200 190 180 170 160 150 140 130 120 110 100 90 80 70 60 50 40 30 20 10 0 -10 -20
f1 (ppm)

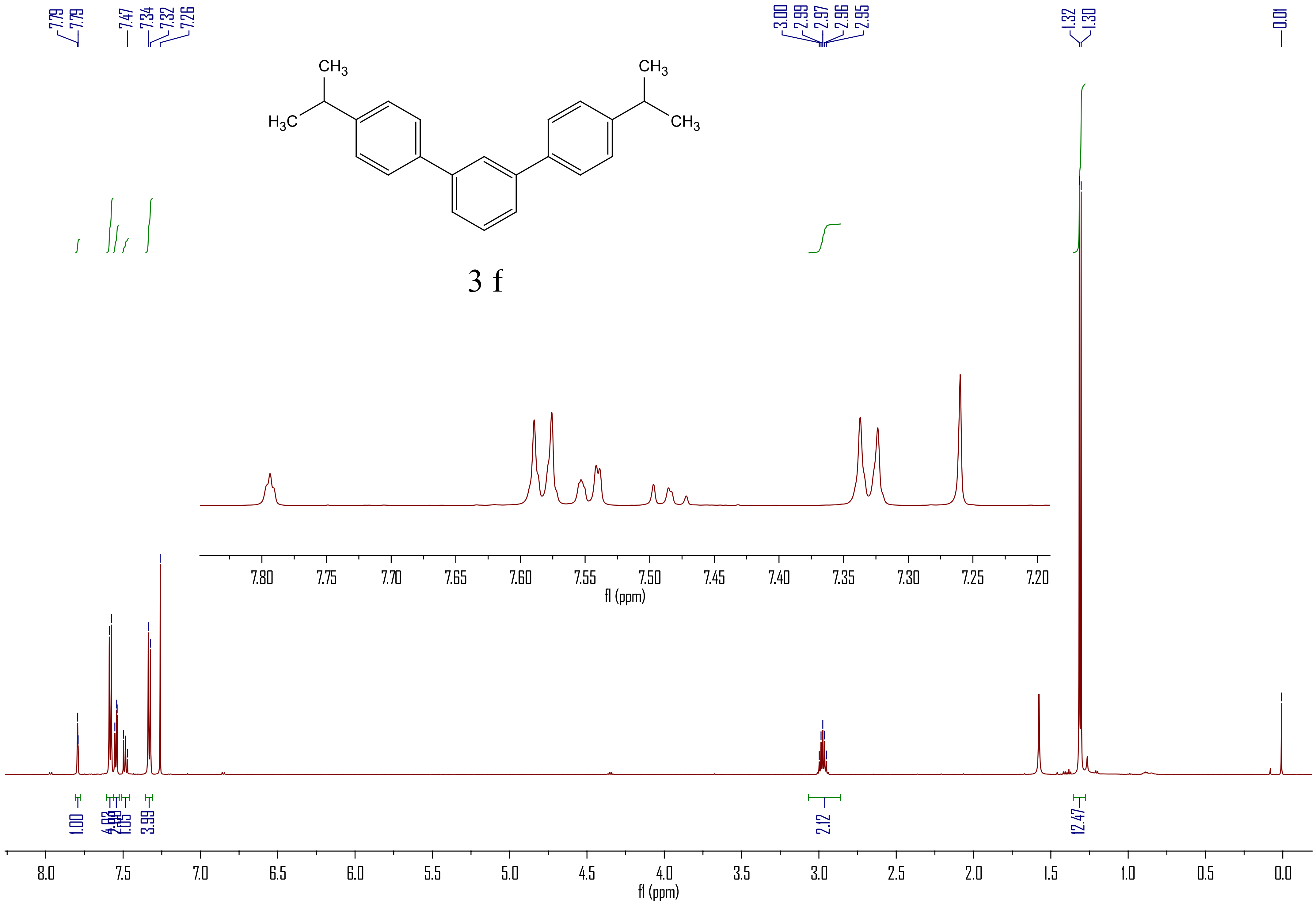

7.79
7.79
7.47
7.34
7.32
7.26
3.00
2.99
2.97
2.96
2.95
1.32
1.30
0.01
CH3
H3C
CH3
CH3
3 f
7.80
7.75
7.70
7.65
7.60
7.55
7.50
7.45
7.40
7.35
7.30
7.25
7.20
fl (ppm)
1.00
4.03
2.09
1.05
3.99
2.12
12.47
8.0
7.5
7.0
6.5
6.0
5.5
5.0
4.5
4.0
3.5
3.0
2.5
2.0
1.5
1.0
0.5
0.0
fl (ppm)

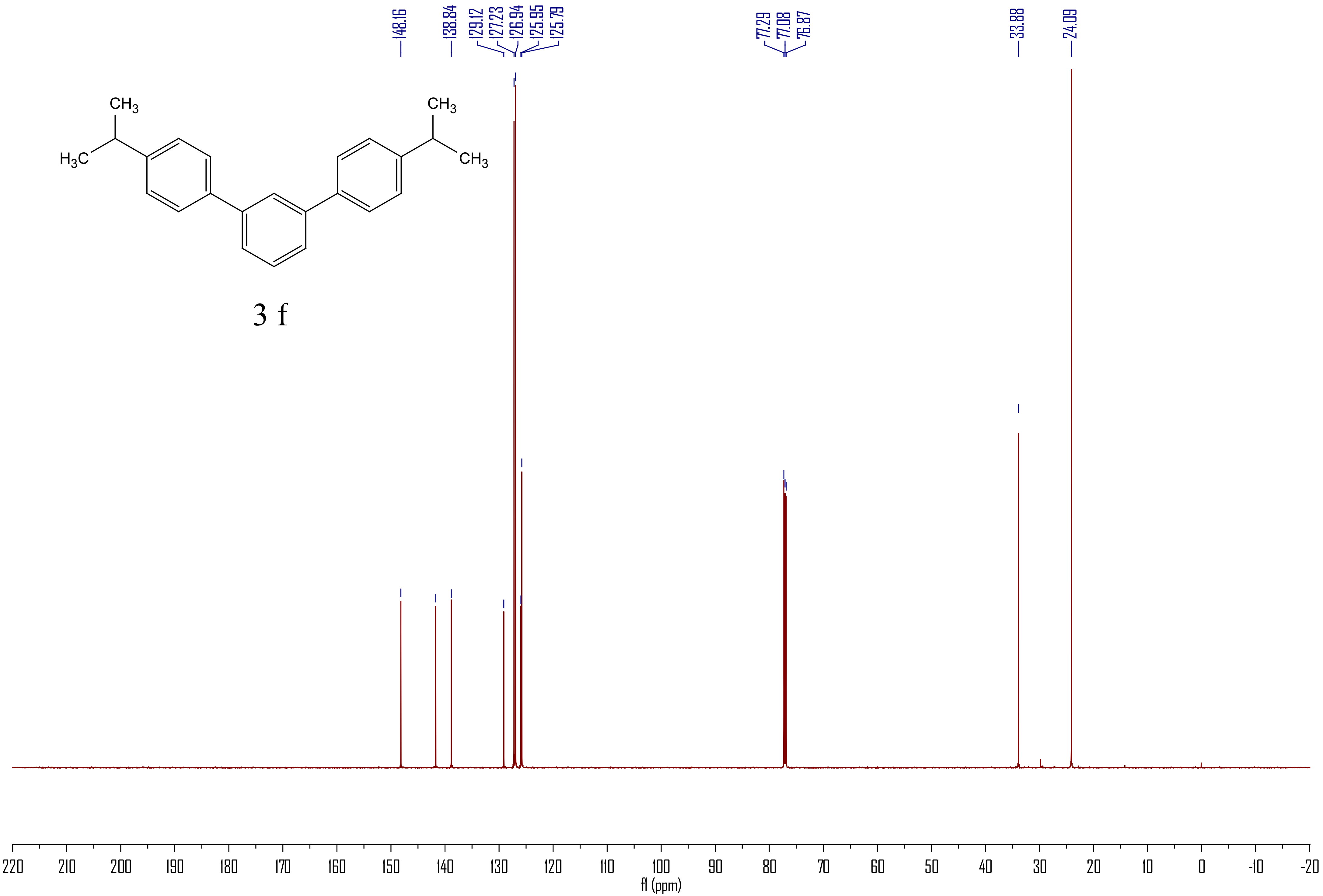
148.16
138.84
129.12
127.23
126.94
125.95
125.79
77.29
77.08
76.87
33.88
24.09
CH3
H3C
CH3
CH3
3 f
220 210 200 190 180 170 160 150 140 130 120 110 100 90 80 70 60 50 40 30 20 10 0 -10 -20
f1 (ppm)

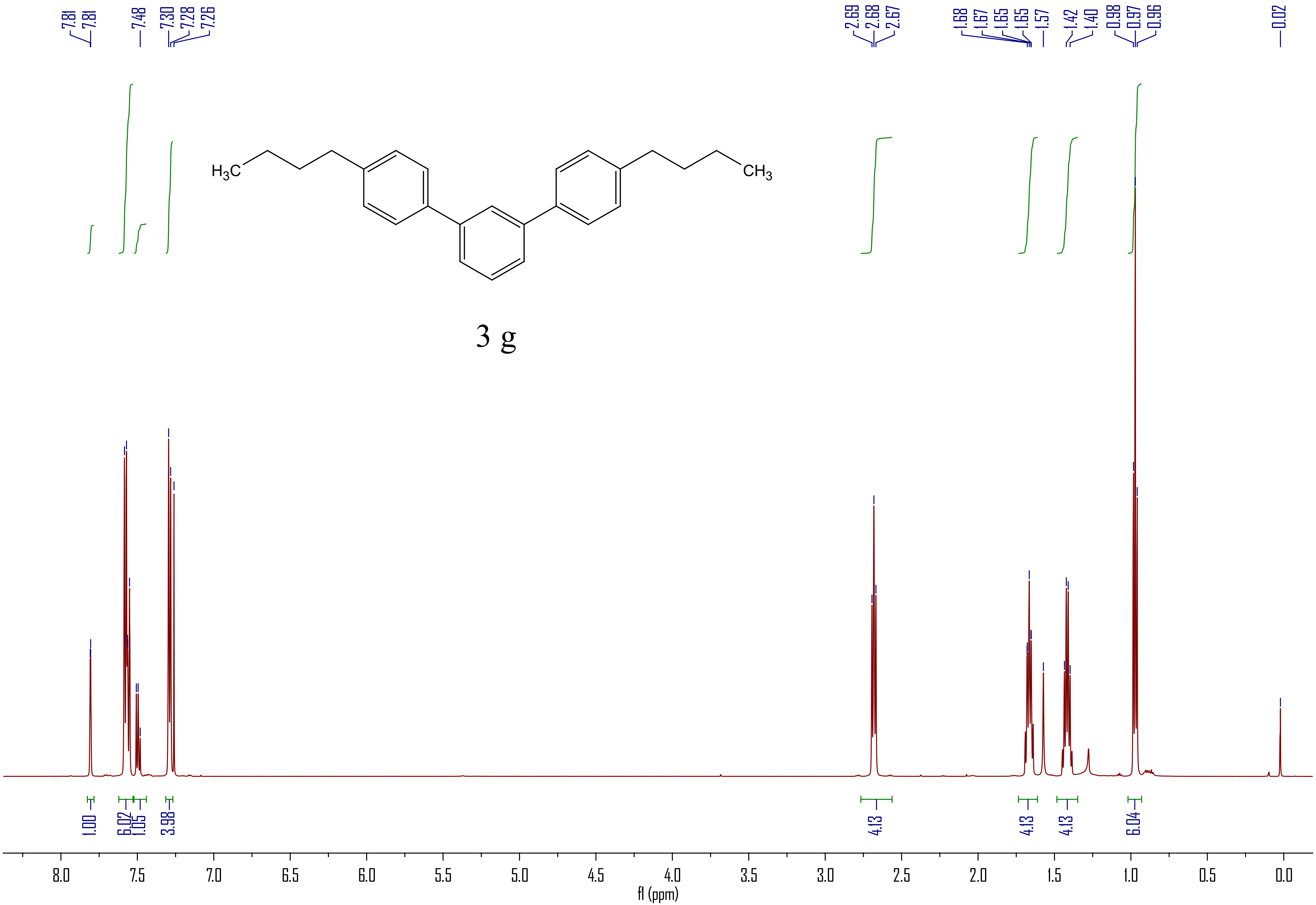
7.81
7.81
7.48
7.30
7.28
7.26
2.69
2.68
2.67
1.68
1.67
1.65
1.65
1.57
1.42
1.40
0.98
0.97
0.96
0.02
H_3C
CH_3
3 g
1.00
6.02
1.05
3.98
4.13
4.13
4.13
6.04
8.0
7.5
7.0
6.5
6.0
5.5
5.0
4.5
4.0
3.5
3.0
2.5
2.0
1.5
1.0
0.5
0.0
f1 (ppm)

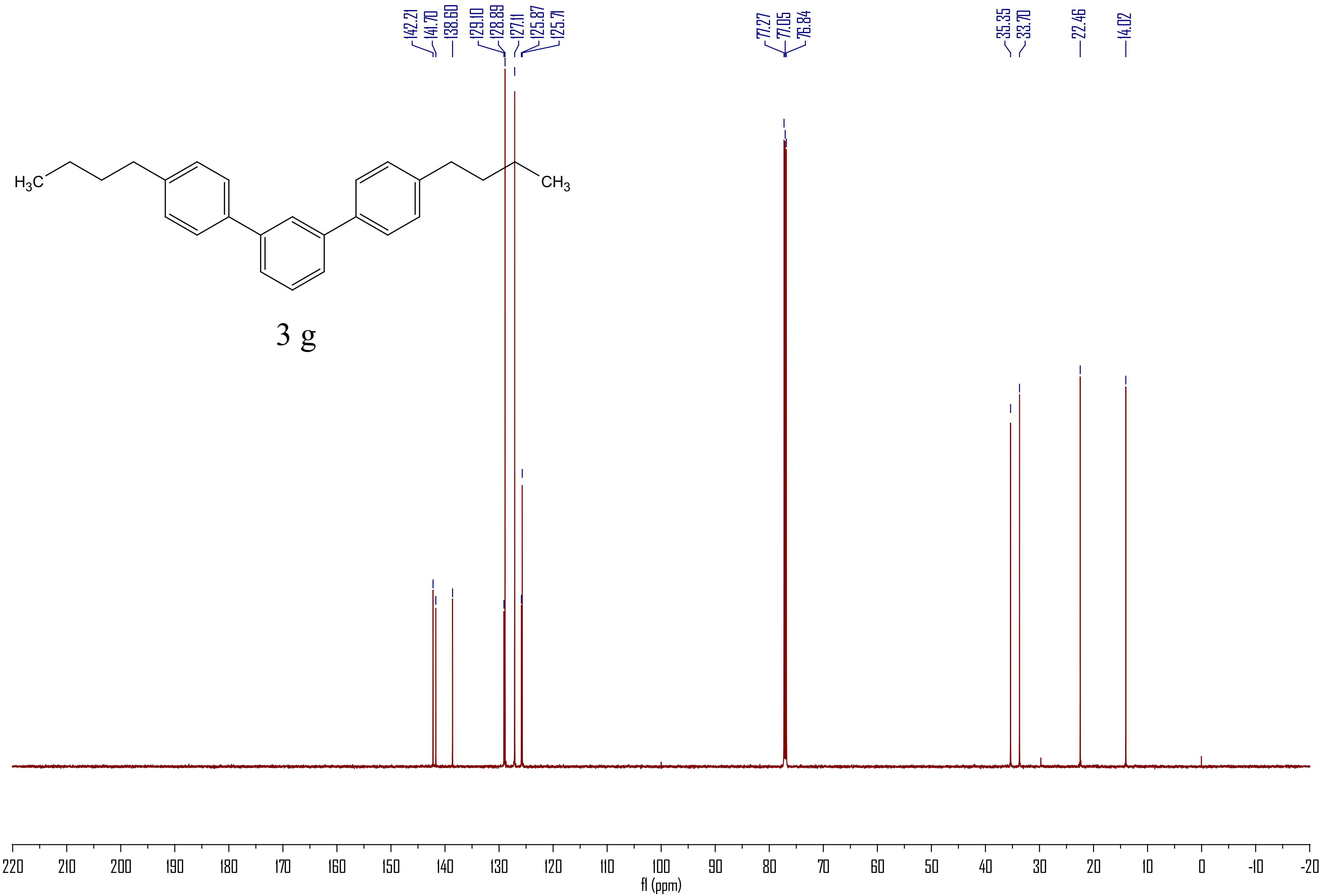
142.21
141.70
138.60
129.10
128.89
127.11
125.87
125.71
77.27
77.05
76.84
35.35
33.70
22.46
14.02
H_3C
CH_3
220 210 200 190 180 170 160 150 140 130 120 110 100 90 80 70 60 50 40 30 20 10 0 -10 -20
f1 (ppm)

3 g

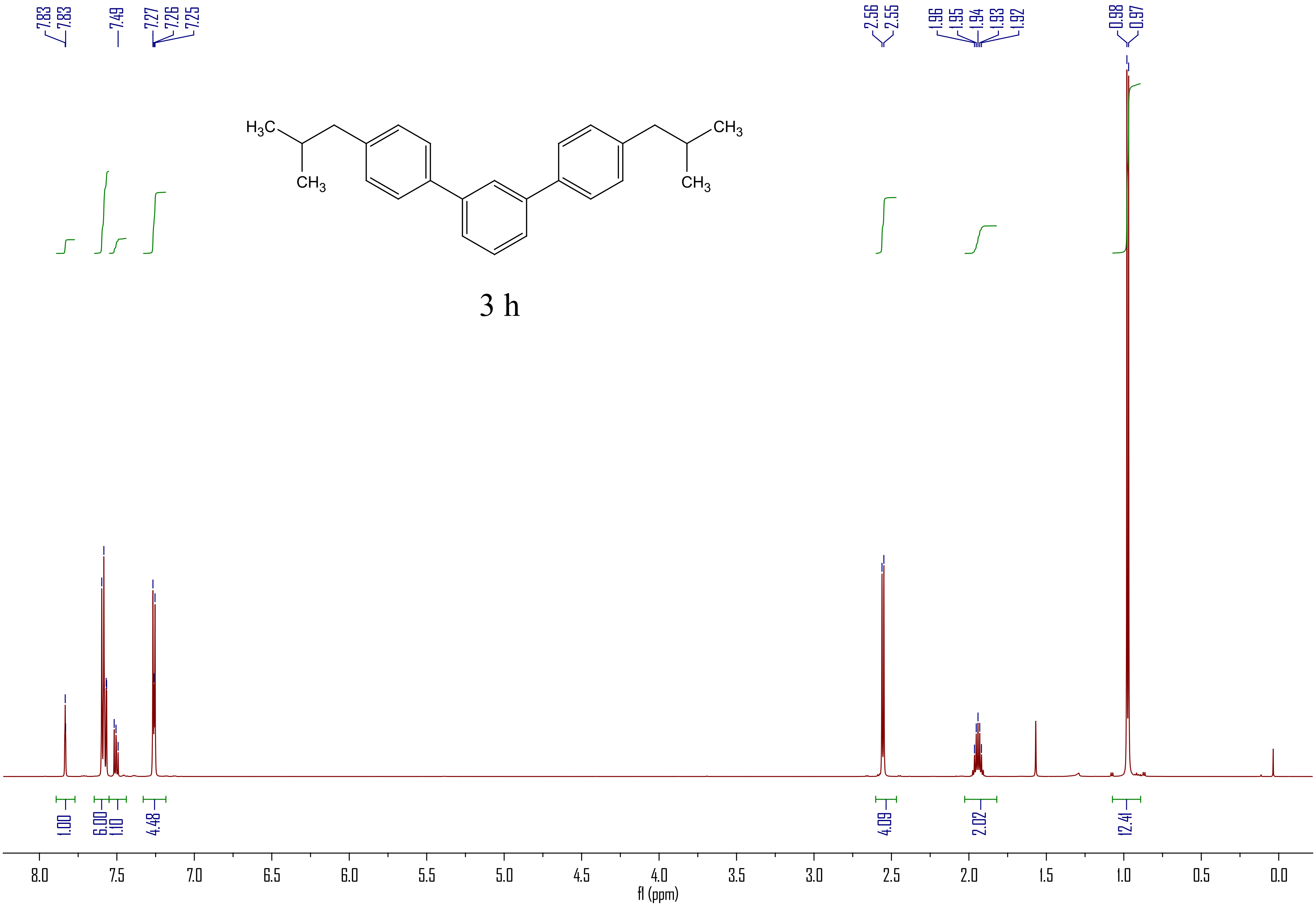
7.83
7.83
7.49
7.27
7.26
7.25
2.56
2.55
1.96
1.95
1.94
1.93
1.92
0.98
0.97
H_3C
CH_3
CH_3
CH_3
1.00
6.00
1.10
4.48
4.09
2.02
12.41
8.0
7.5
7.0
6.5
6.0
5.5
5.0
4.5
4.0
3.5
3.0
2.5
2.0
1.5
1.0
0.5
0.0
f1 (ppm)

3 h

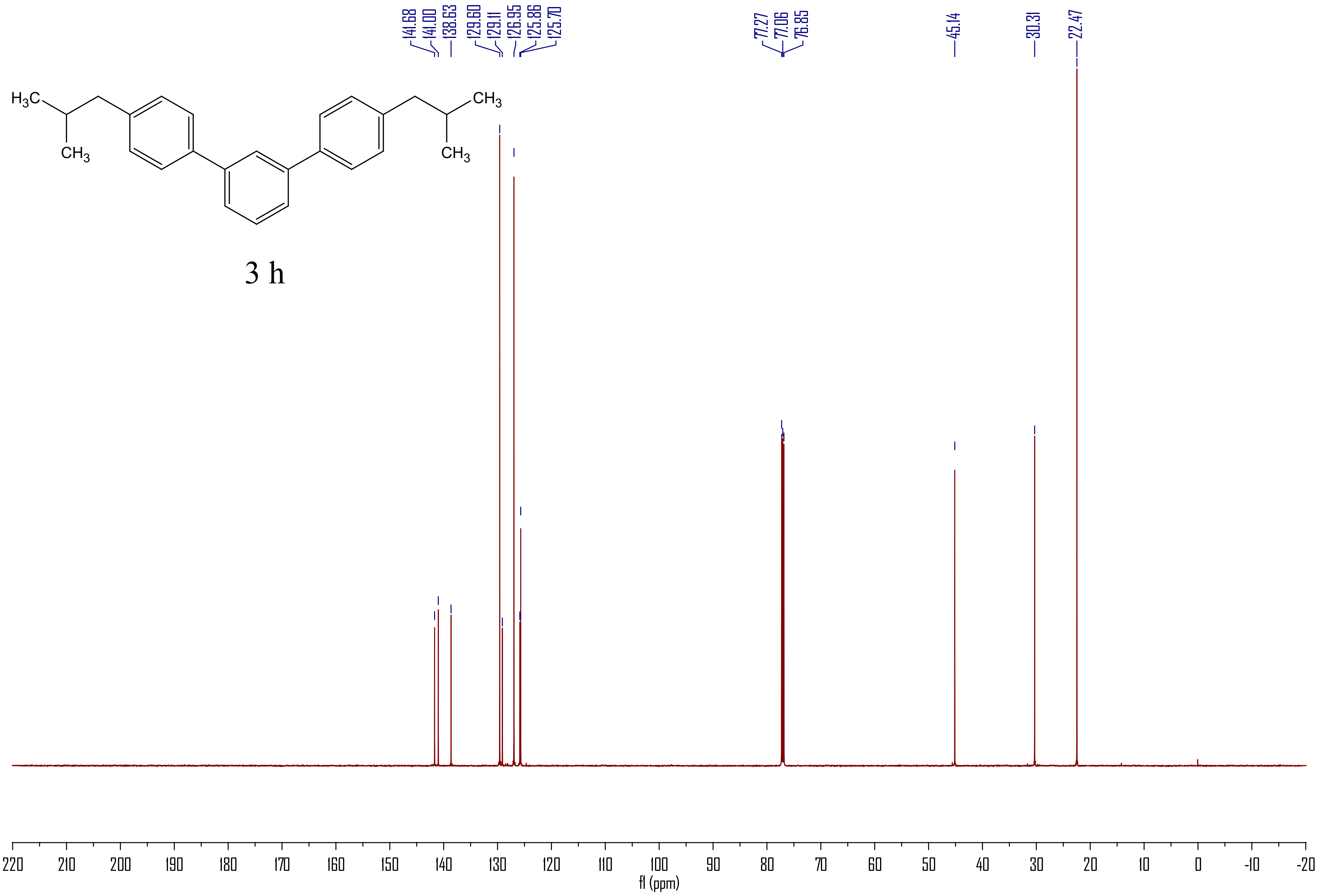
141.68
141.00
138.63
129.60
129.11
126.95
125.86
125.70
77.27
77.06
76.85
45.14
30.31
22.47
H3C
CH3
CH3
CH3
220 210 200 190 180 170 160 150 140 130 120 110 100 90 80 70 60 50 40 30 20 10 0 -10 -20
f1 (ppm)

3 h

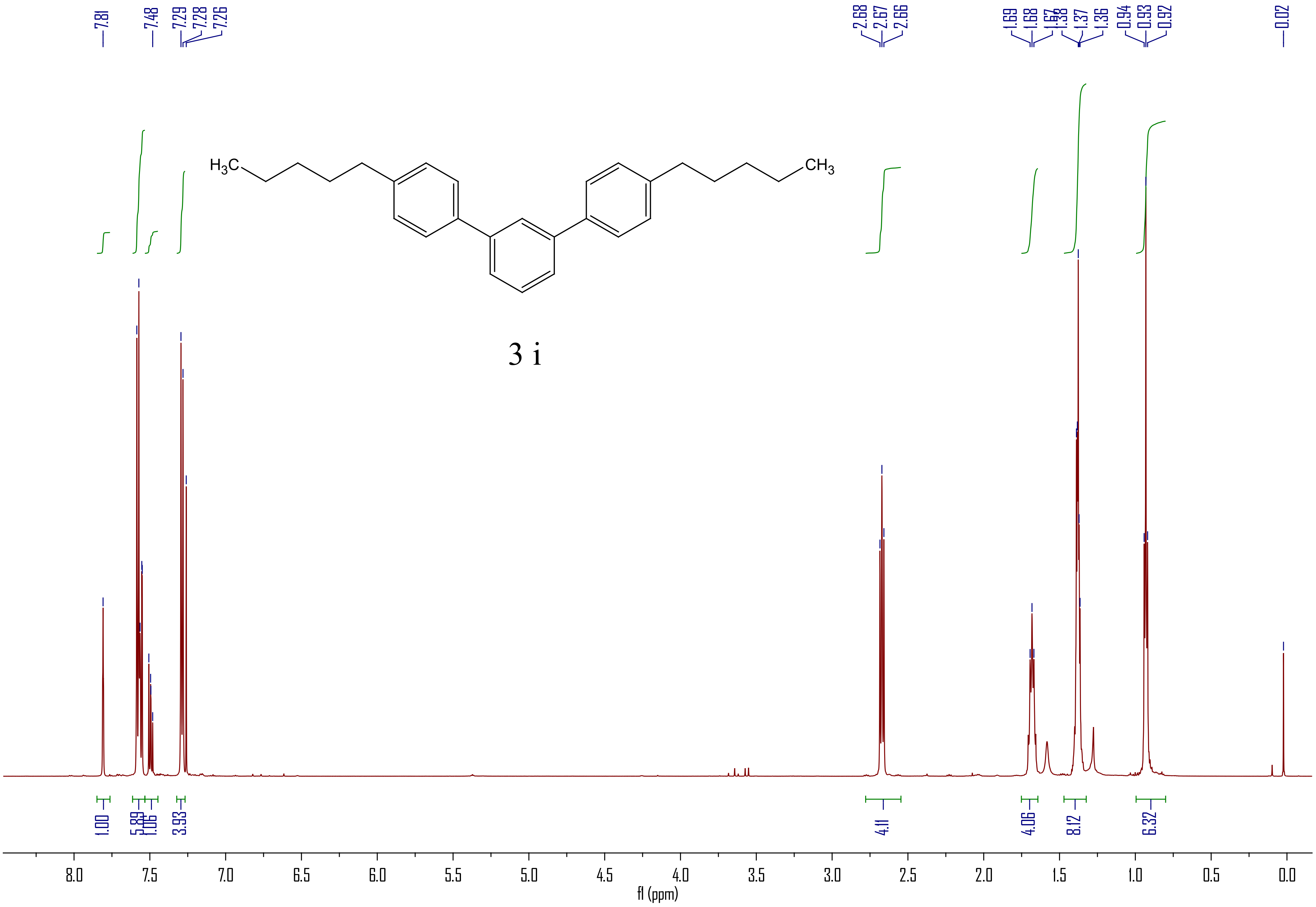
7.81
7.48
7.29
7.28
7.26
2.68
2.67
2.66
1.69
1.68
1.38
1.37
1.36
0.94
0.93
0.92
0.02
H3C
CH3
3 i
1.00
5.89
1.06
3.93
4.11
4.06
8.12
6.32
8.0
7.5
7.0
6.5
6.0
5.5
5.0
4.5
4.0
3.5
3.0
2.5
2.0
1.5
1.0
0.5
0.0
f1 (ppm)

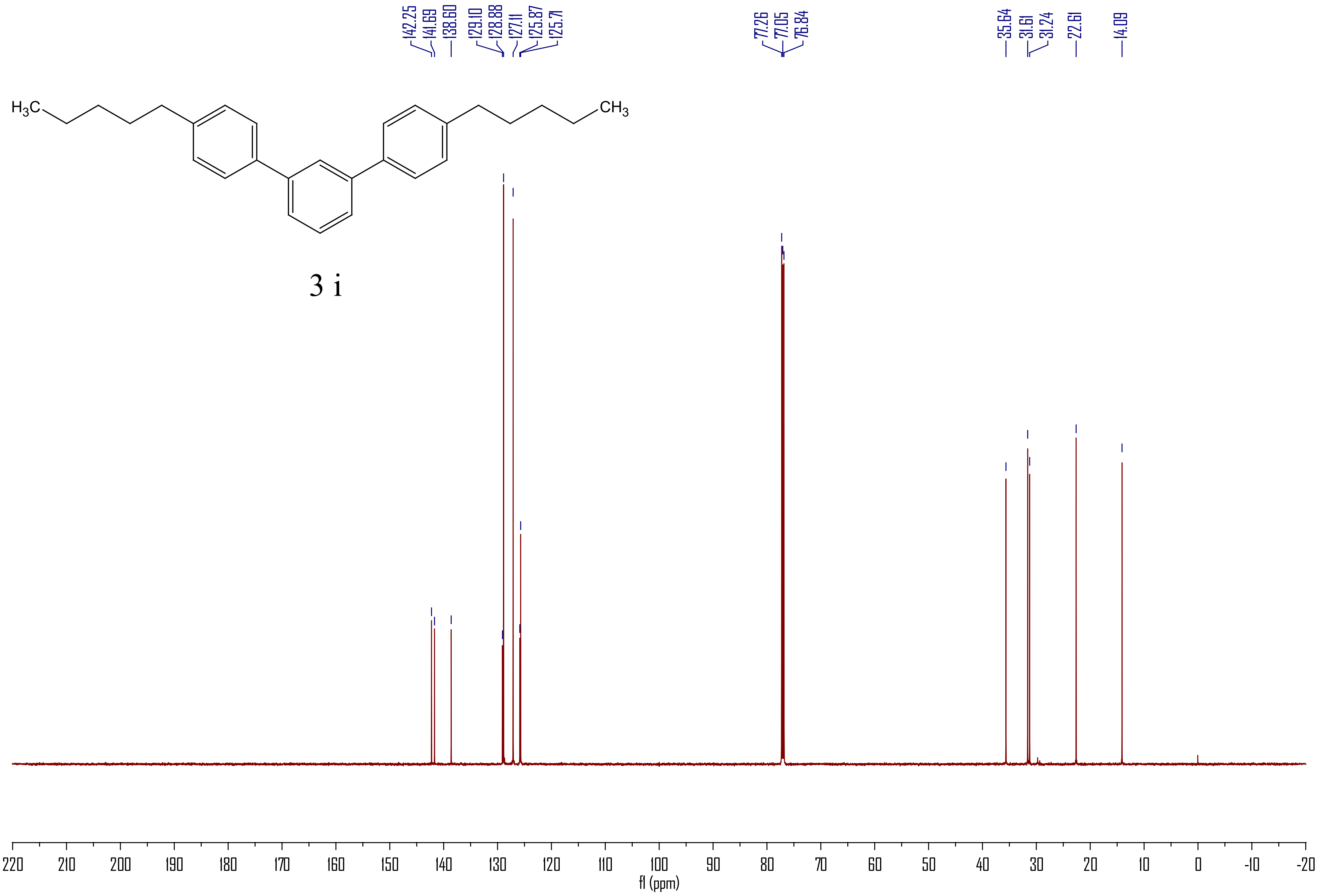
142.25
141.69
138.60
129.10
128.88
127.11
125.87
125.71
77.26
77.05
76.84
35.64
31.61
31.24
22.61
14.09
H3C
CH3
3 i
220 210 200 190 180 170 160 150 140 130 120 110 100 90 80 70 60 50 40 30 20 10 0 -10 -20
f1 (ppm)

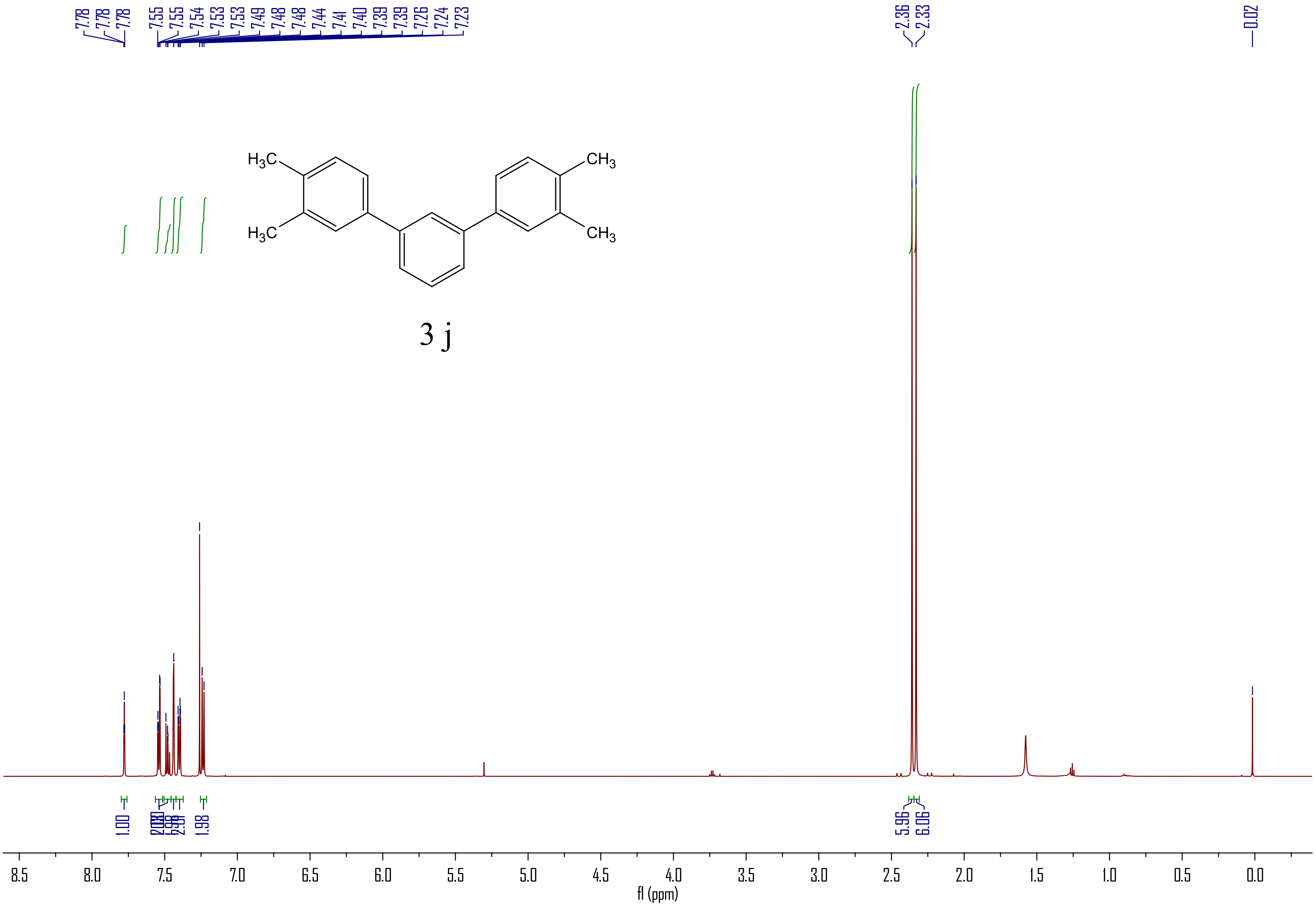
7.78 7.78 7.78
7.55 7.55 7.54 7.53 7.53 7.49 7.48 7.48 7.44 7.41 7.40 7.39 7.39 7.26 7.24 7.23
2.36 2.33
0.02
H3C
H3C
CH3
CH3
3 j
1.00
1.98
5.96
6.06
8.5 8.0 7.5 7.0 6.5 6.0 5.5 5.0 4.5 4.0 3.5 3.0 2.5 2.0 1.5 1.0 0.5 0.0
f1 (ppm)

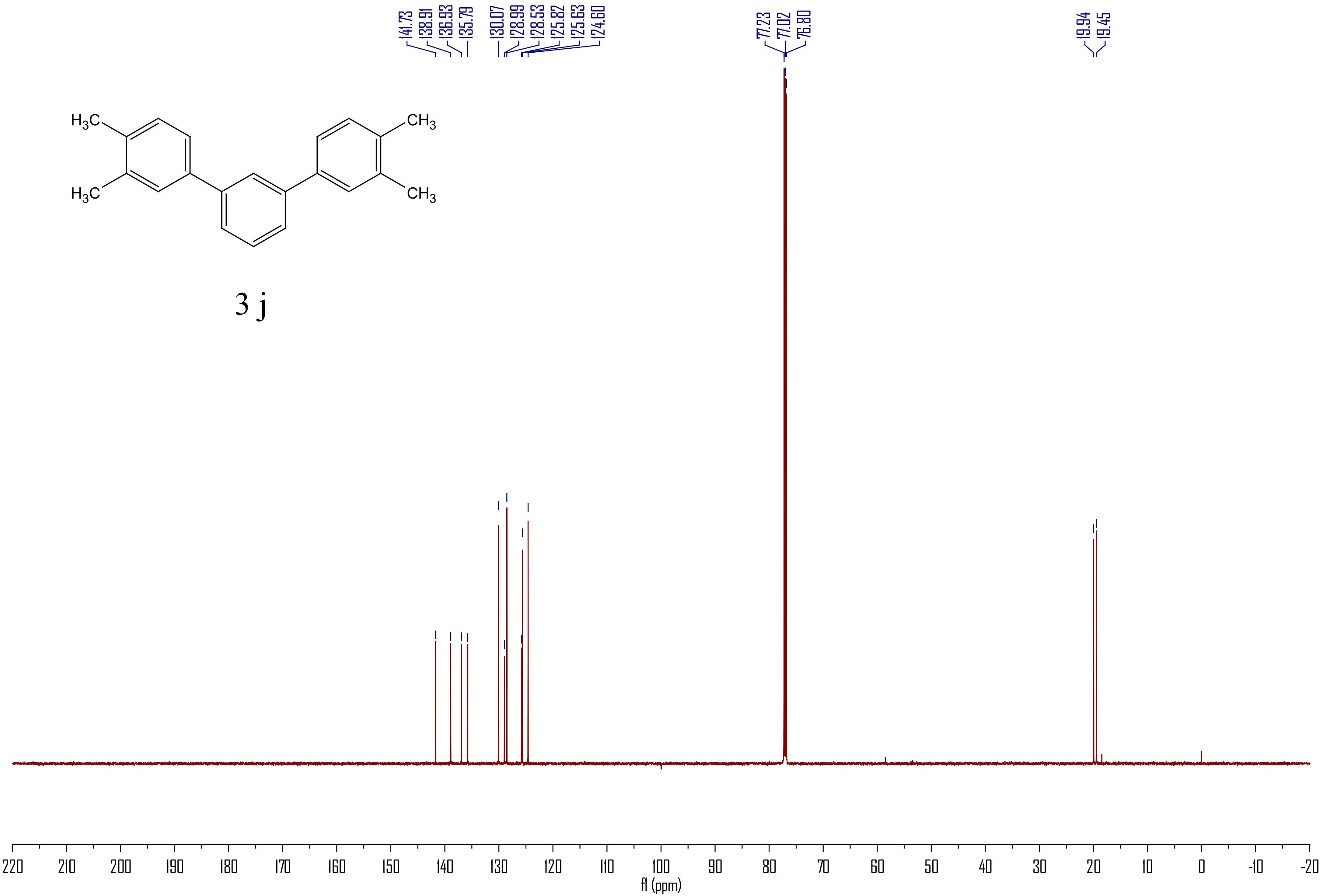
141.73
138.91
136.93
135.79
130.07
128.99
128.53
125.82
125.63
124.60
77.23
77.02
76.80
19.94
19.45
H3C
CH3
H3C
CH3
3 j
220 210 200 190 180 170 160 150 140 130 120 110 100 90 80 70 60 50 40 30 20 10 0 -10 -20
f1 (ppm)

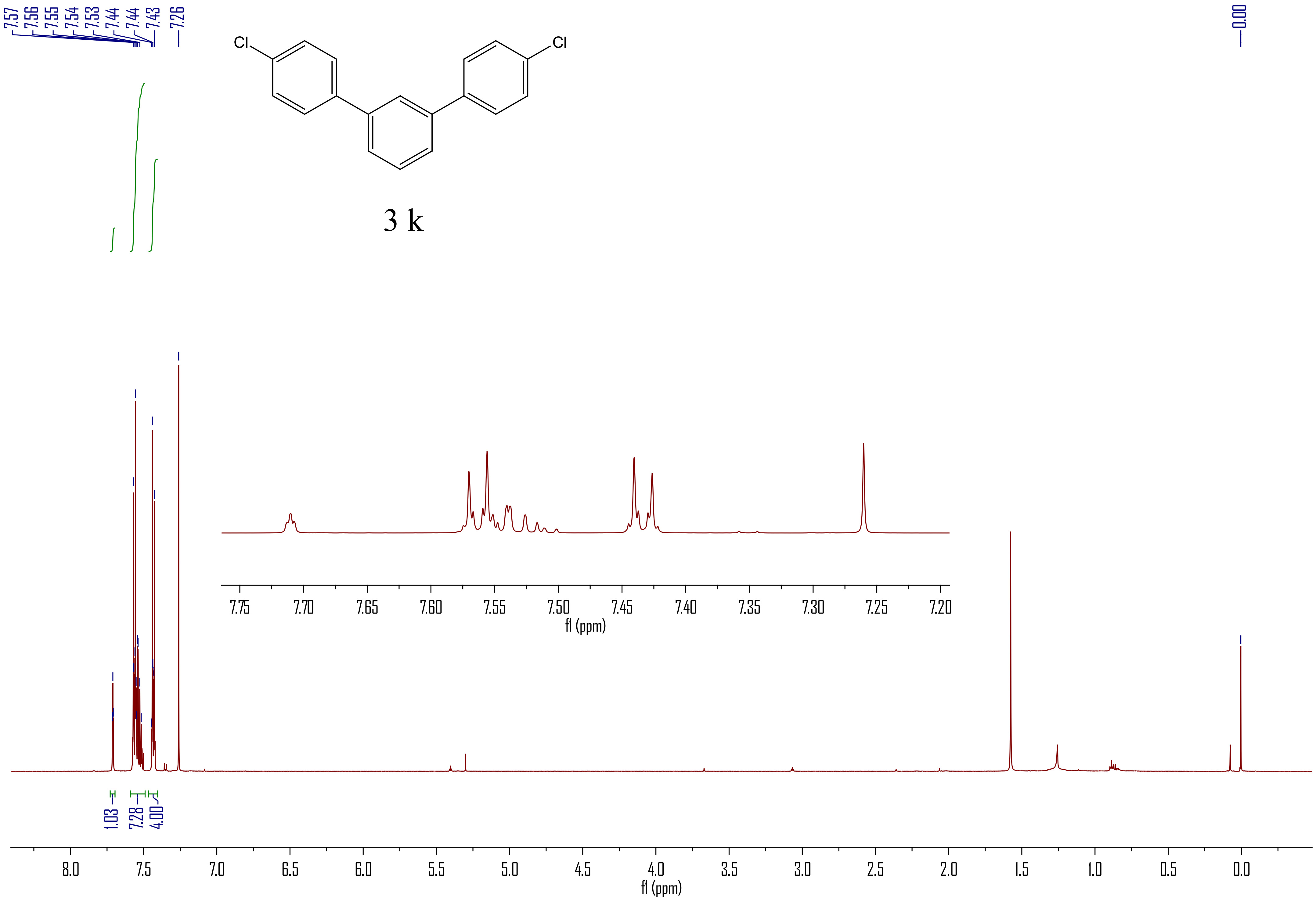
7.57
7.56
7.55
7.54
7.53
7.44
7.44
7.43
7.26
0.00
Cl
Cl
3 k
1.03
7.28
4.00
fl (ppm)
fl (ppm)

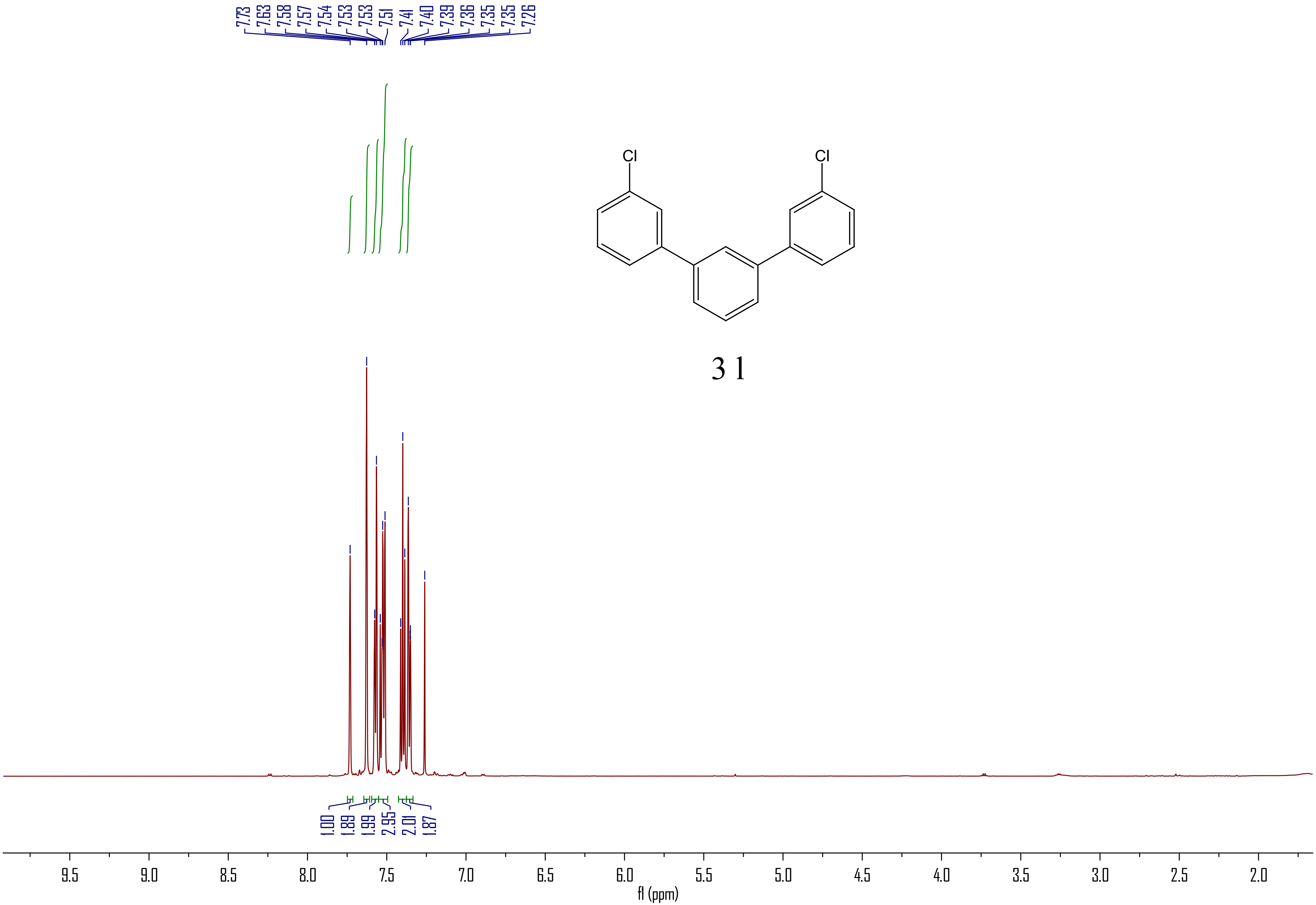
7.73
7.63
7.58
7.57
7.54
7.53
7.53
7.51
7.41
7.40
7.39
7.36
7.35
7.35
7.35
7.26
Cl
Cl
3 l
1.00
1.89
1.99
2.95
2.01
1.87
9.5
9.0
8.5
8.0
7.5
7.0
6.5
6.0
5.5
5.0
4.5
4.0
3.5
3.0
2.5
2.0
f1 (ppm)

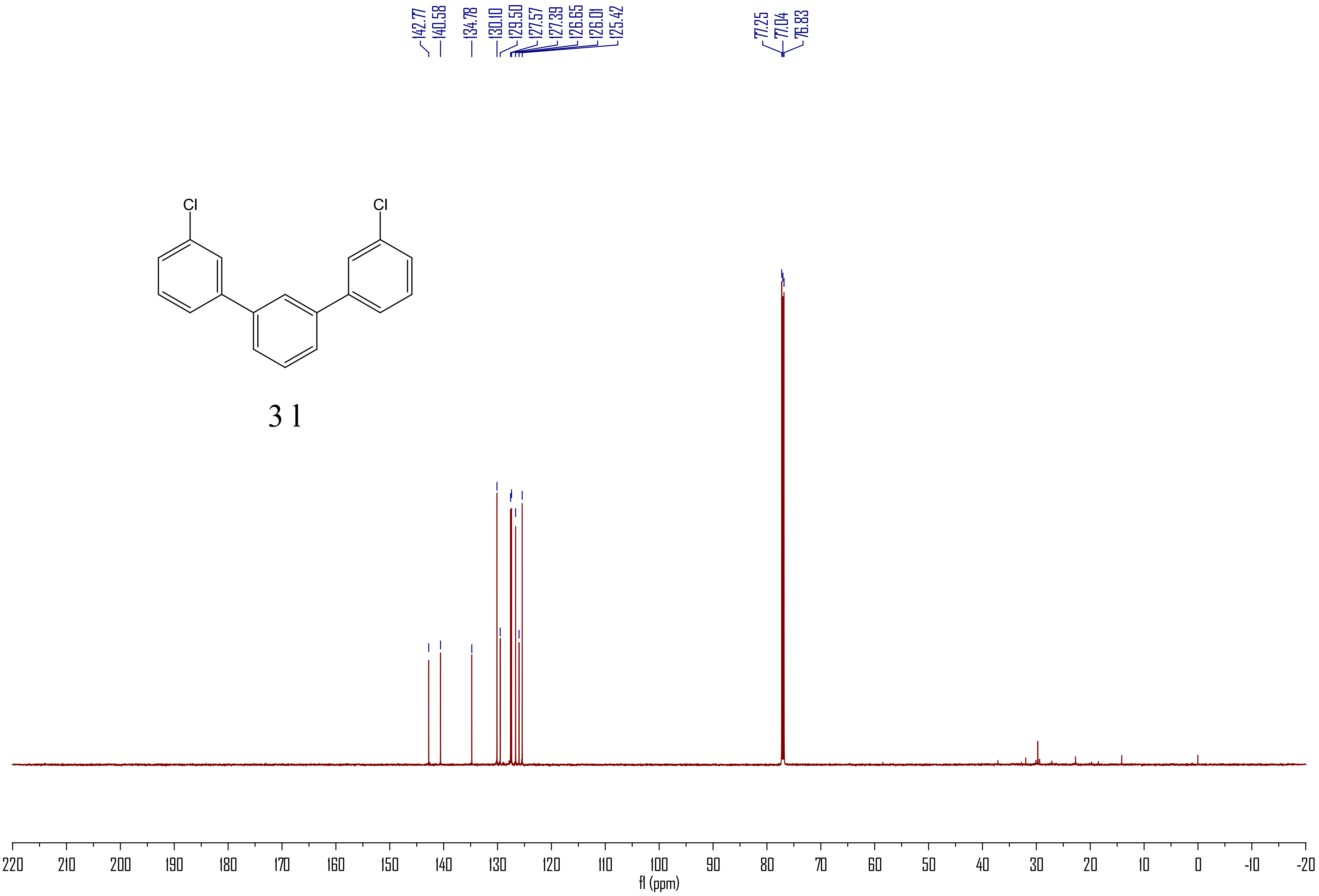
142.77
140.58
134.78
130.10
129.50
127.57
127.39
126.65
126.01
125.42
77.25
77.04
76.83
Cl
Cl
220
210
200
190
180
170
160
150
140
130
120
110
100
90
80
70
60
50
40
30
20
10
0
-10
-20
f1 (ppm)

31

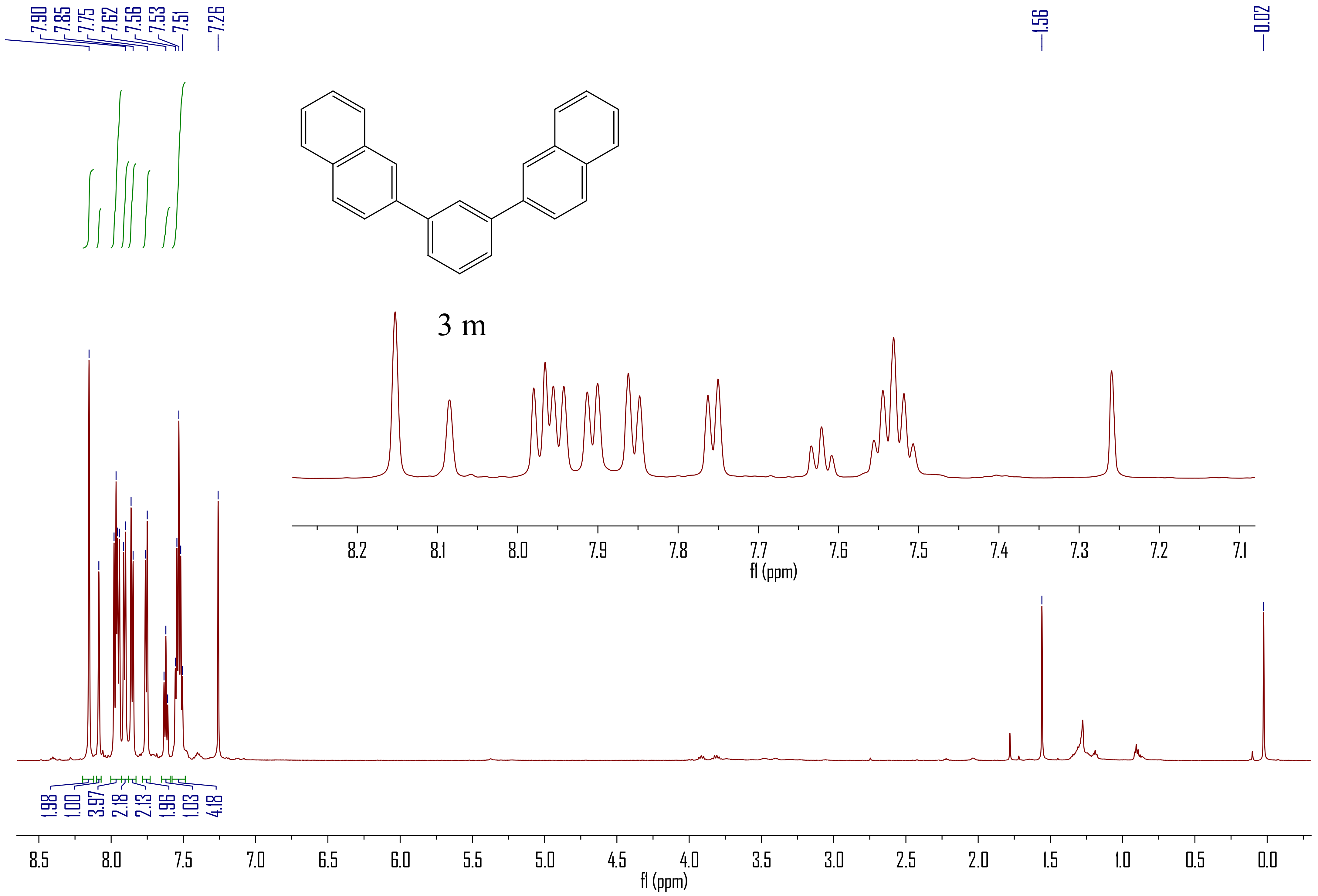
7.90
7.85
7.75
7.62
7.56
7.53
7.51
7.26
1.56
0.02
3 m
8.2 8.1 8.0 7.9 7.8 7.7 7.6 7.5 7.4 7.3 7.2 7.1
f1 (ppm)
1.98
1.00
3.97
2.18
2.13
1.96
1.03
4.18
8.5 8.0 7.5 7.0 6.5 6.0 5.5 5.0 4.5 4.0 3.5 3.0 2.5 2.0 1.5 1.0 0.5 0.0
f1 (ppm)

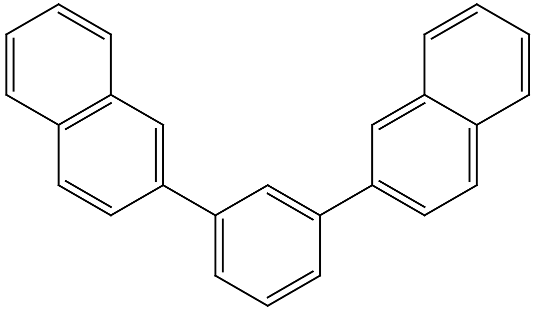

3 m

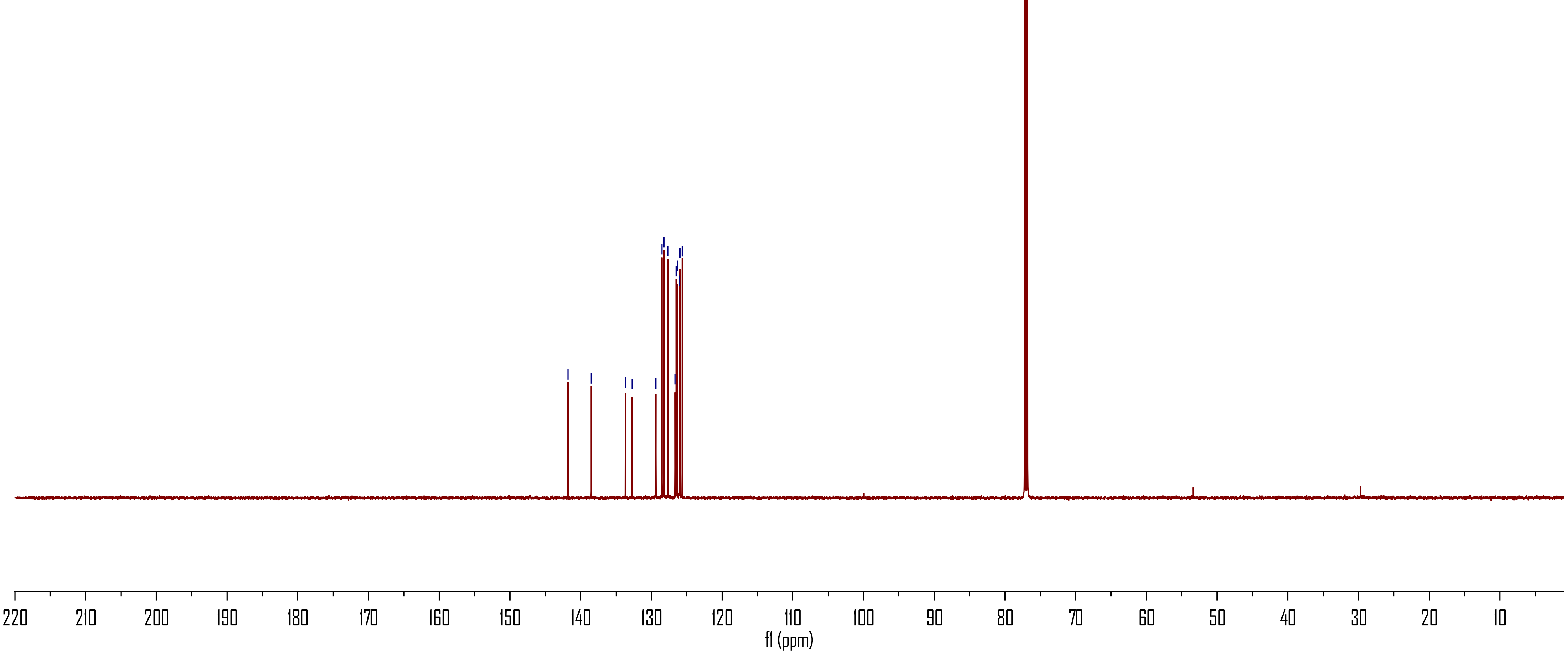
141.80
138.50
128.51
128.23
127.67
126.64
126.48
126.36
126.02
125.97
125.65
77.22
77.01
76.80
220 210 200 190 180 170 160 150 140 130 120 110 100 90 80 70 60 50 40 30 20 10
f1 (ppm)

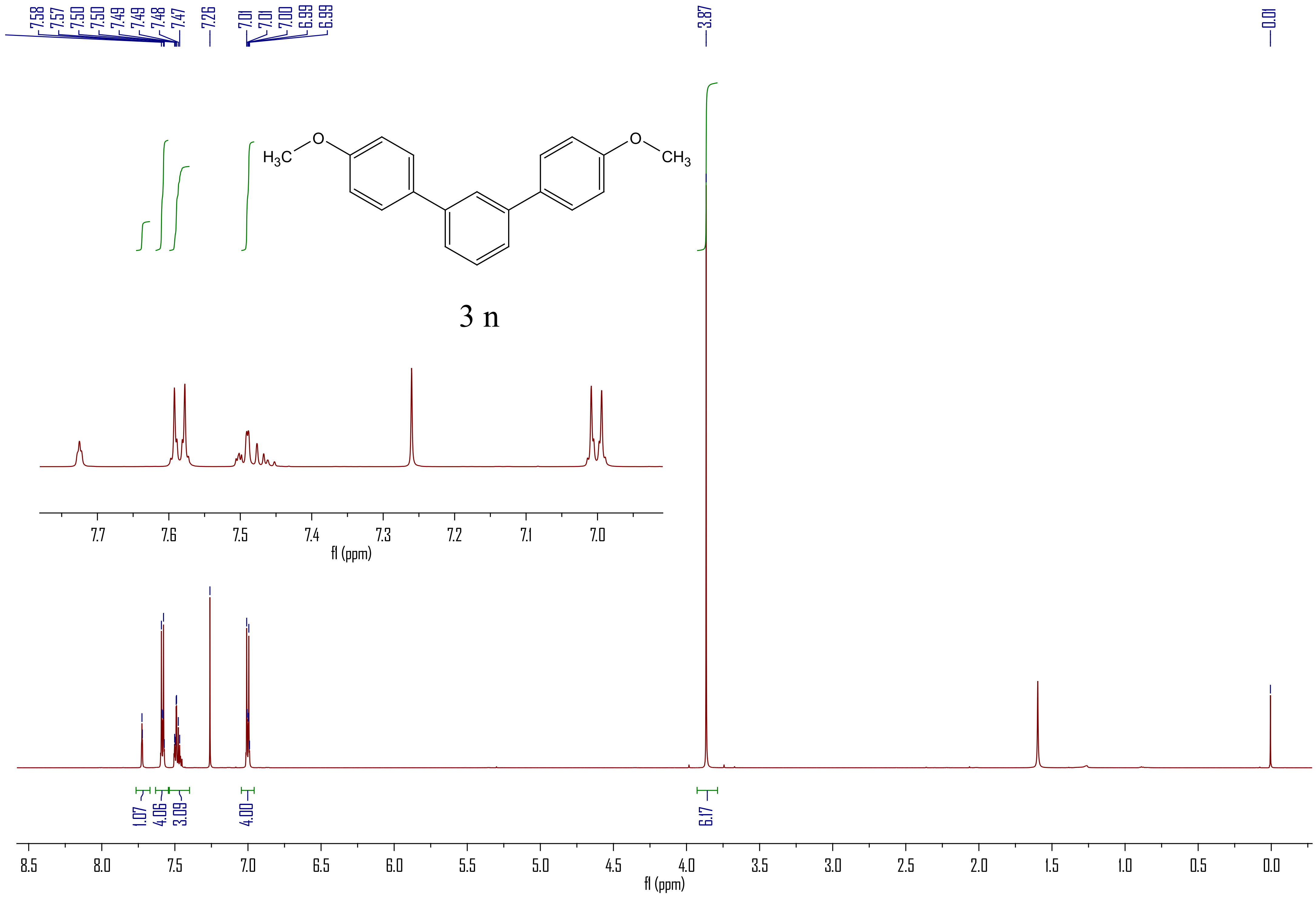
7.58
7.57
7.50
7.50
7.49
7.49
7.48
7.47
7.26
7.01
7.01
7.00
6.99
6.99
3.87
0.01
H_3C
O
O
CH_3
3 n
7.7
7.6
7.5
7.4
7.3
7.2
7.1
7.0
f1 (ppm)
1.07
4.06
3.09
4.00
6.17
8.5
8.0
7.5
7.0
6.5
6.0
5.5
5.0
4.5
4.0
3.5
3.0
2.5
2.0
1.5
1.0
0.5
0.0
f1 (ppm)

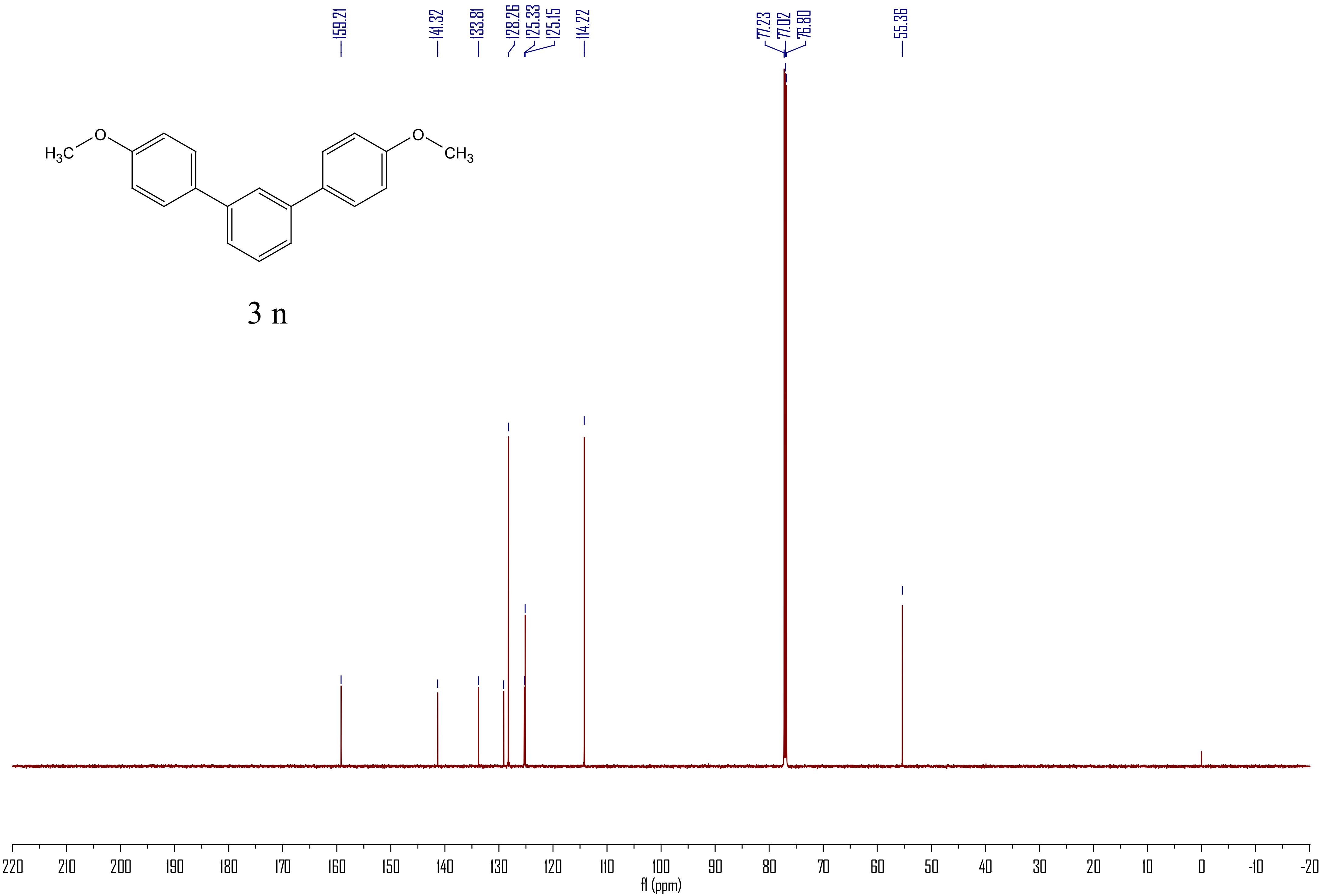
159.21
141.32
133.81
128.26
125.33
125.15
114.22
77.23
77.02
76.80
55.36
H3C
O
O
CH3
3 n
220 210 200 190 180 170 160 150 140 130 120 110 100 90 80 70 60 50 40 30 20 10 0 -10 -20
f1 (ppm)

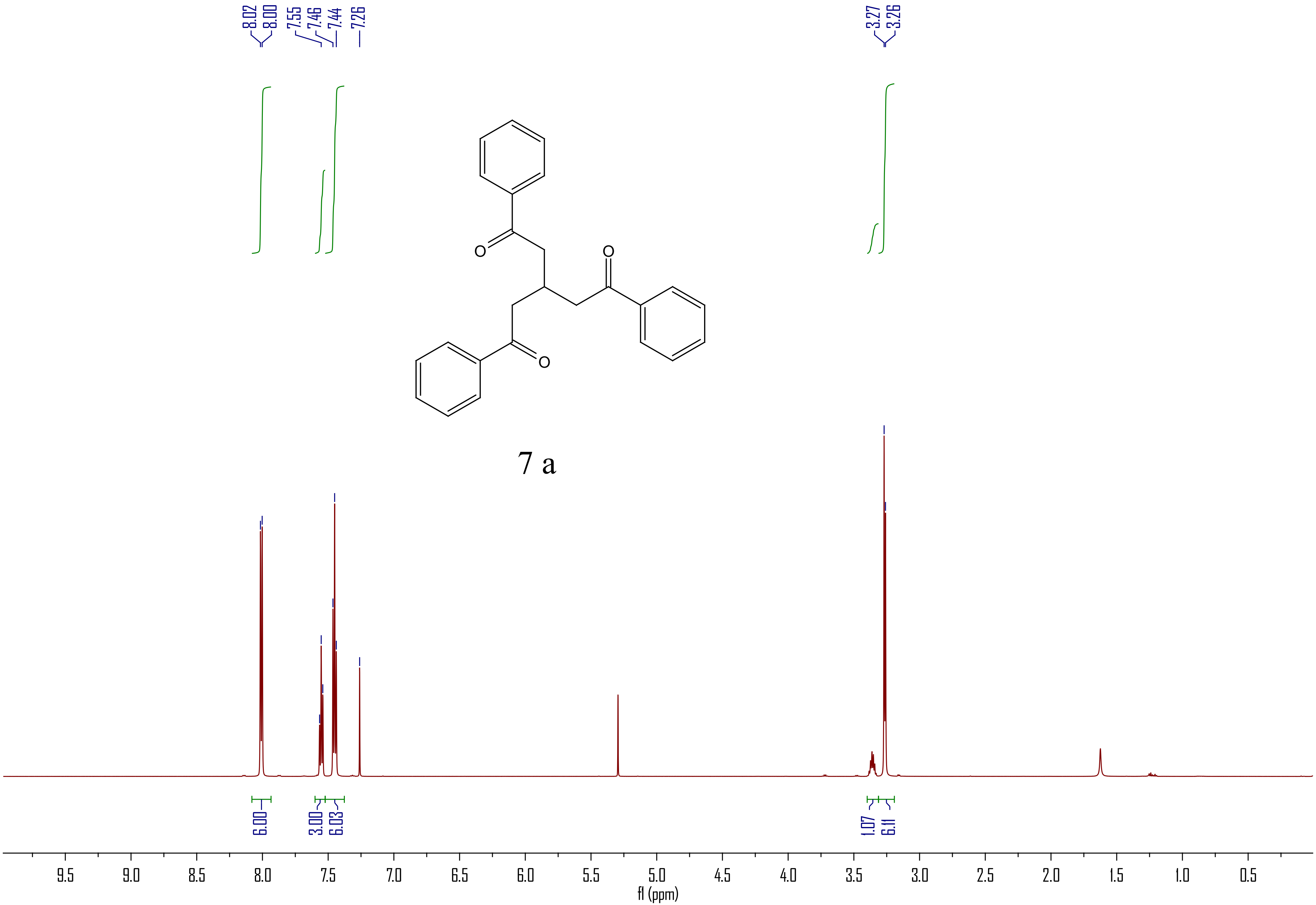
8.02
8.00
7.55
7.46
7.44
7.26
3.27
3.26
7 a
6.00
3.00
6.03
1.07
6.11
9.5 9.0 8.5 8.0 7.5 7.0 6.5 6.0 5.5 5.0 4.5 4.0 3.5 3.0 2.5 2.0 1.5 1.0 0.5
f1 (ppm)

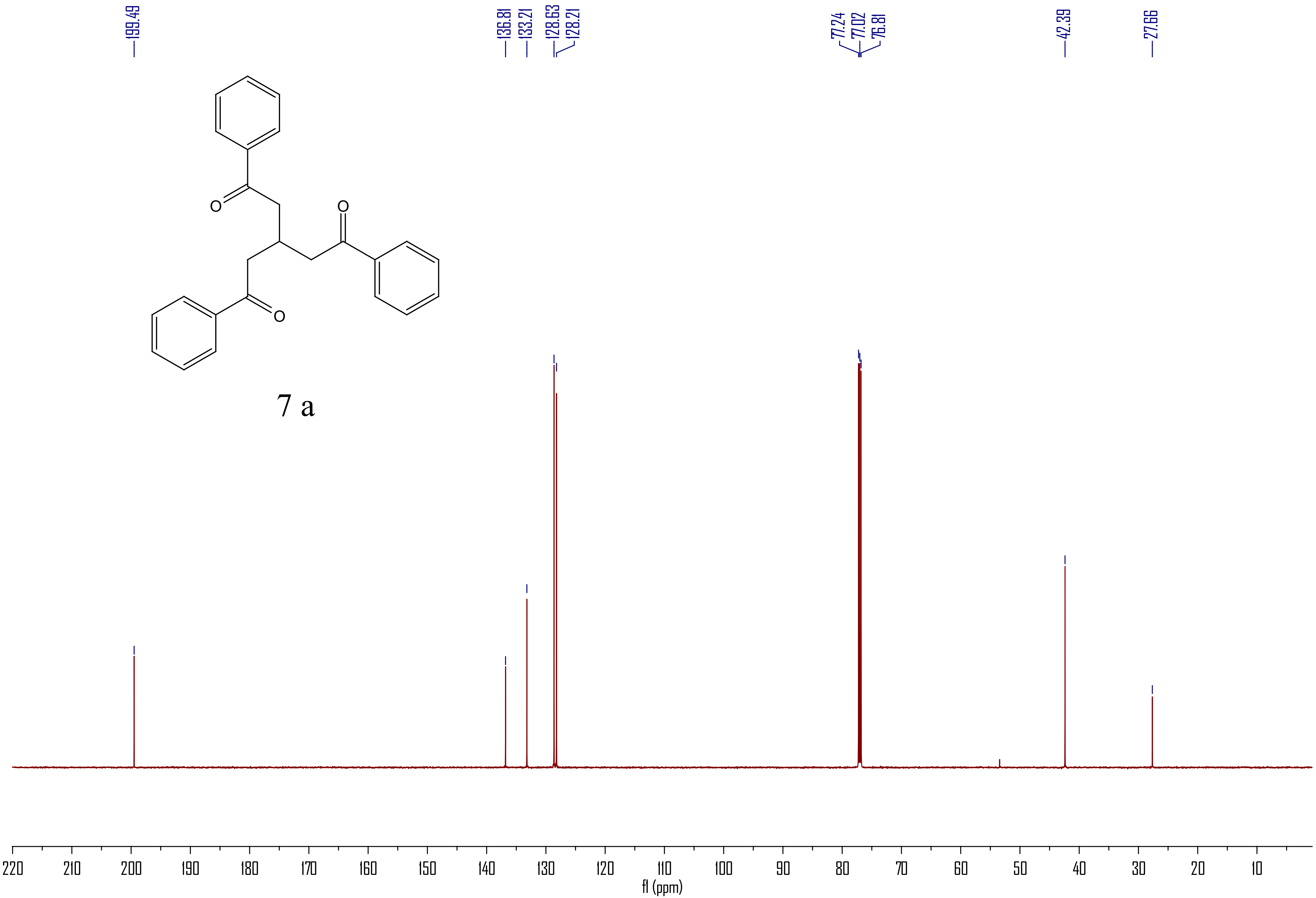

199.49
136.81
133.21
128.63
128.21
77.24
77.02
76.81
42.39
27.66
O
O
O
7 a
220
210
200
190
180
170
160
150
140
130
120
110
100
90
80
70
60
50
40
30
20
10
f1 (ppm)

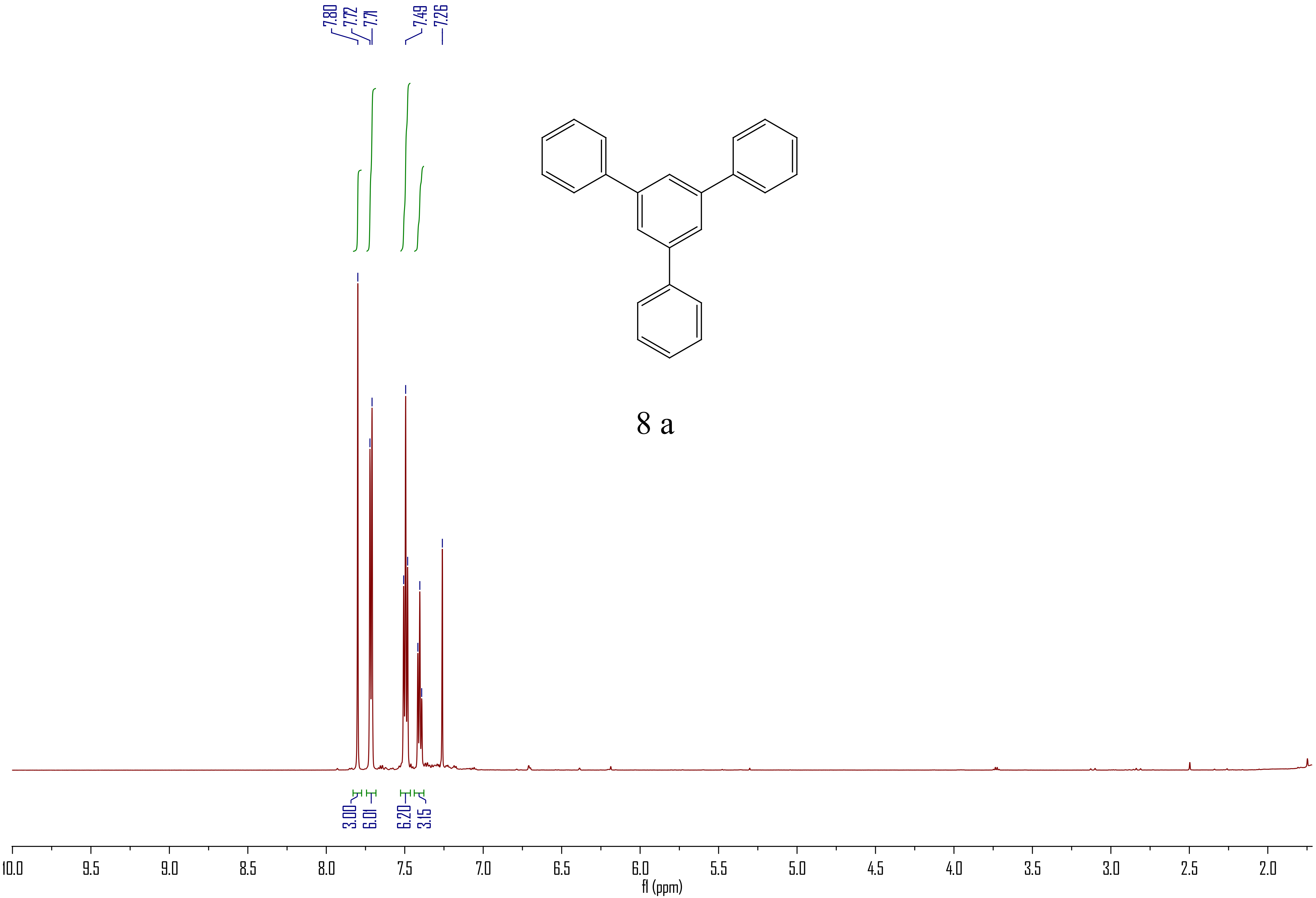
7.80
7.72
7.71
7.49
7.26
8 a
3.00
6.01
6.20
3.15
10.0
9.5
9.0
8.5
8.0
7.5
7.0
6.5
6.0
5.5
5.0
4.5
4.0
3.5
3.0
2.5
2.0
f1 (ppm)

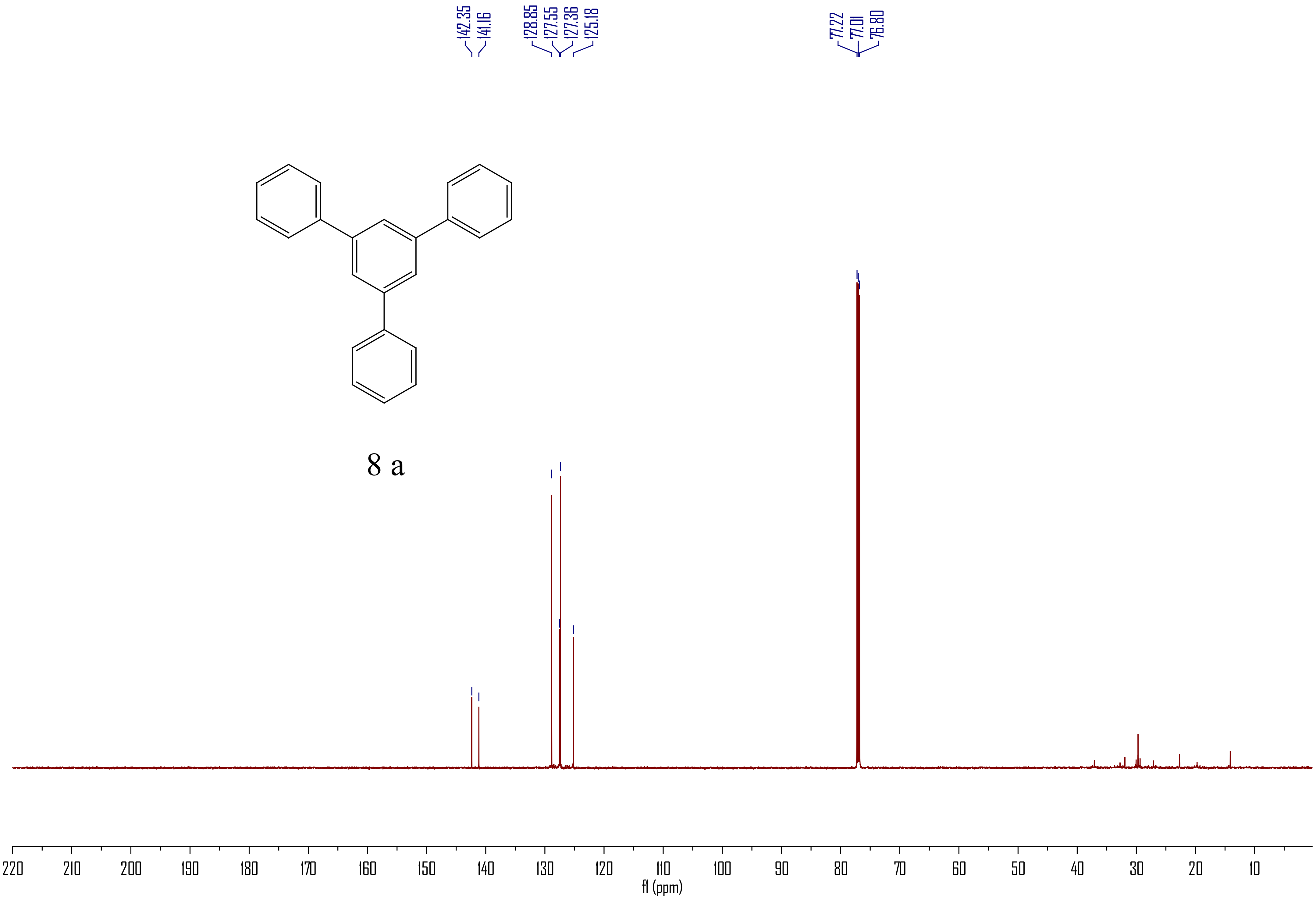
142.35
141.16
128.85
127.55
127.36
125.18
77.22
77.01
76.80
8 a
220
210
200
190
180
170
160
150
140
130
120
110
100
90
80
70
60
50
40
30
20
10
f1 (ppm)

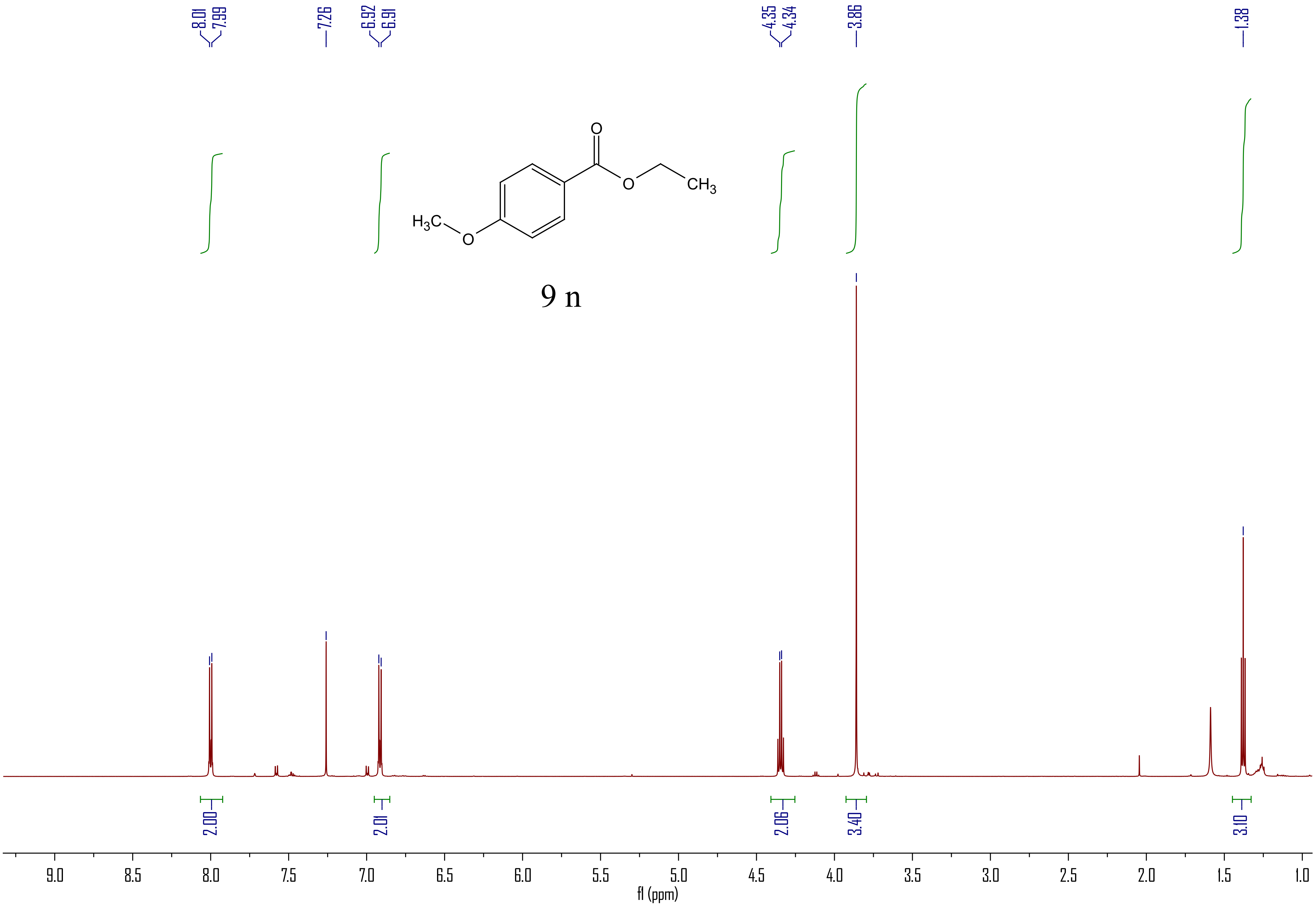
8.01
7.99
7.26
6.92
6.91
4.35
4.34
3.86
1.38
O
O
H3C
O
CH3
2.00
2.01
2.06
3.40
3.10
9.0
8.5
8.0
7.5
7.0
6.5
6.0
5.5
5.0
4.5
4.0
3.5
3.0
2.5
2.0
1.5
1.0
f1 (ppm)

9 n

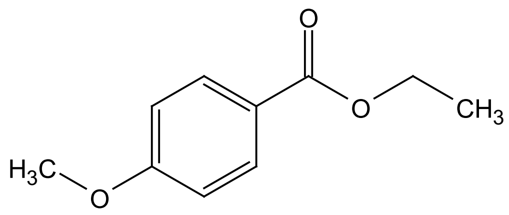

9 n

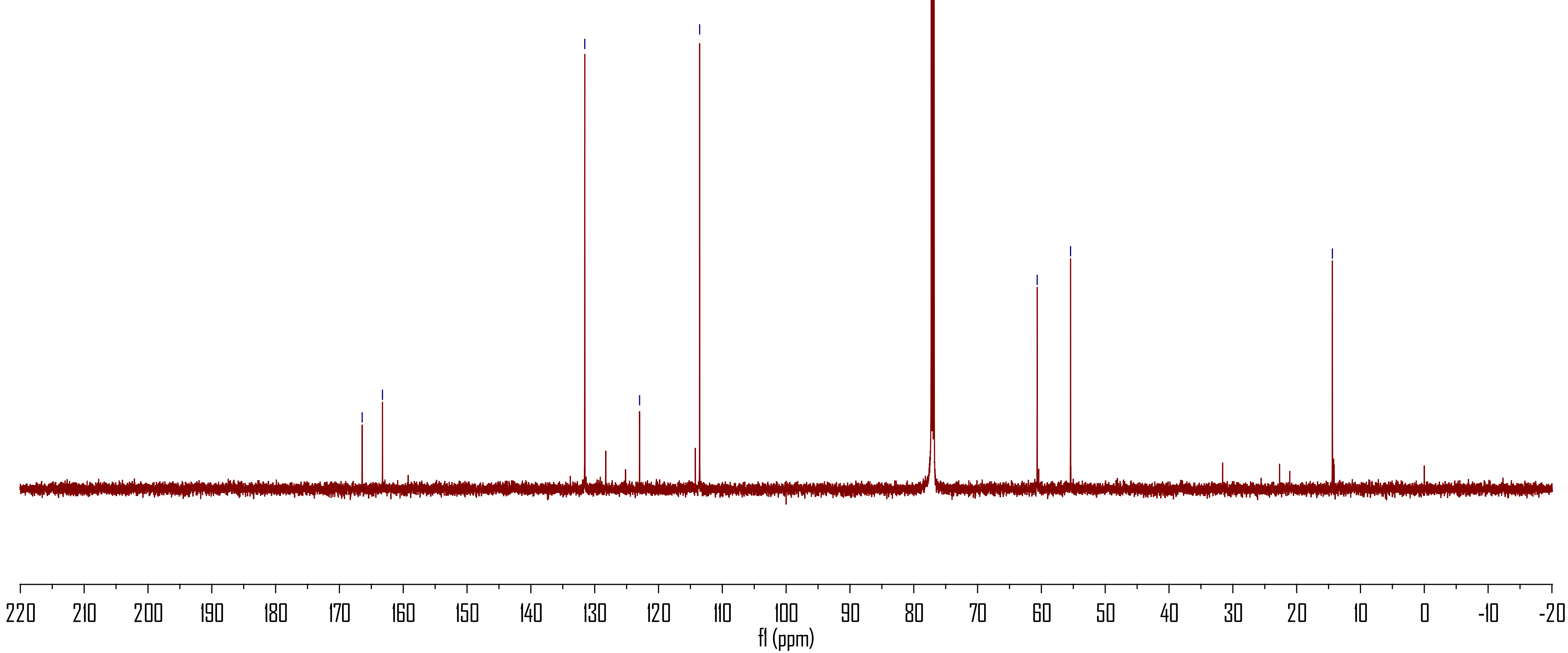
166.43
163.25
131.55
122.97
113.55
77.25
77.03
76.82
60.66
55.43
14.40
220 210 200 190 180 170 160 150 140 130 120 110 100 90 80 70 60 50 40 30 20 10 0 -10 -20
f1 (ppm)
